# An Eulerian evaluation of intense low-pressure systems over North America in CMIP6 and a regional climate model

**DOI:** 10.1007/s00382-026-08260-7

**Published:** 2026-06-26

**Authors:** Victorien De Meyer, Alejandro Di Luca, Philippe Gachon

**Affiliations:** 1 Centre Pour l’Étude et la Simulation du Climat à l’Échelle Régionale (ESCER), Université du Québec à Montréal, Montréal, QC H2X 3Y7 Canada; 2 Department of Earth and Atmospheric Sciences, Université du Québec à Montréal, Montréal, QC H2X 3Y7 Canada; 3 Department of Geography, Université du Québec à Montréal, Montréal, QC H2X 3Y7 Canada

**Keywords:** Extratropical cyclones, Climate model evaluation, Regional climate models (RCMs), Dynamical downscaling

## Abstract

Low-pressure systems, such as extratropical cyclones (ETCs), are a key component of mid-latitude climate variability and frequent drivers of extreme weather in North America. This study evaluates 27 CMIP6 global climate models (GCMs) and the Canadian Regional Climate Model (CRCM6), driven by ERA5 reanalysis or three CMIP6 GCMs, in reproducing surface pressure anomalies and near-surface humidity during intense cyclonic events over 1980–2014. We introduce a novel Eulerian approach that identifies the strongest low-pressure systems at each grid point and characterizes their local temporal evolution using a three-parameter decomposition: $$peak $$ intensity, mean $$tendency $$, and $$asymmetry $$. This decomposition effectively captures key regional differences and provides a new approach to assess model biases in simulating local dynamical and thermodynamical conditions associated with intense systems. Results indicate that GCMs share systematic biases in characterizing local environmental changes, though the origins vary regionally. Model resolution influences error magnitude but is less critical over continental areas. In high track density regions or areas affected by explosive cyclones, higher resolution reduces pressure anomaly errors, while near-surface humidity shows no clear resolution-related pattern. CRCM6 simulations exhibit the smallest errors domain-wide and perform consistently regardless of driving data. They show particular added value north of $$45^{\circ }$$N and over oceans, especially where GCM errors are large, although some substantial errors persist in certain regions. This study underscores the challenges in simulating intense extratropical and tropical cyclones, highlights the importance of accurately representing synoptic and regional surface conditions, and provides a framework for evaluating model performance, informing improved dynamical downscaling and climate projections.

**Supplementary Information:**

The online version contains supplementary material available at 10.1007/s00382-026-08260-7.

## Introduction

Low-pressure systems encompass a wide range of atmospheric phenomena, including mesoscale cyclones, extratropical cyclones (ETCs), and tropical cyclones (TCs). Throughout the year, ETCs are the predominant low-pressure systems associated with precipitation across most of North America, with the exception of the tropical Atlantic, where TCs can have a significant impact during the hurricane season (Utsumi et al. 2017). ETCs are large-scale low-pressure systems that occur in mid- to high-latitude regions, capable of traveling thousands of kilometers over several days. They are a fundamental component of the mid-latitude climate variability and play a pivotal role in shaping regional climate dynamics and their associated societal impacts (Seneviratne et al. 2021). These systems develop in regions of strong temperature gradients between polar and tropical air masses (Sanders and Gyakum 1980), contribute up to 90% of total precipitation over major midlatitute storm track regions (Catto et al. 2012; Catto and Pfahl 2013; Hawcroft et al. 2012; Pfahl and Wernli 2012; Utsumi et al. 2017; Zhang and Colle 2017). They are key drivers of extreme weather events, including strong winds and extreme precipitation, which frequently result in significant economic losses due to flooding, landslide, windstorms or coastal storm surge (Browning 2004; Liberato 2014; Chen and Luca 2025; Chen et al. 2022; Dowdy and Catto 2017; Gliksman et al. 2023; Pfahl and Sprenger 2016; Pfahl and Wernli 2012). In addition to their impact on extreme weather, extratropical cyclones are integral to the global hydrological cycle. They are responsible for transporting substantial amounts of moisture and heat, serving as a critical mechanism for redistributing surface water and energy within the atmosphere (Catto et al. 2019; Priestley and Catto 2022). This makes them vital for maintaining the freshwater supply necessary for agriculture and societal needs. Understanding the characteristics and associated dynamics of extratropical cyclones is thus crucial not only for interpreting local and regional climate variability, but also for advancing our ability to monitor and project climate change (Catto et al. 2019; Priestley and Catto 2022; Ulbrich et al. 2009).

Climate models are the primary tools used to investigate changes in extratropical cyclone characteristics under future climate scenarios. Coarse-resolution climate models, here referring to models with horizontal grid spacings typically exceeding 150–200 km, are generally considered capable of capturing the broad features of mid-latitude storm tracks (Priestley et al. 2020; Priestley and Catto 2022; Flato et al. 2014). However, they are subject to numerous limitations. ETCs simulated by coarse-resolution models often exhibit weaker intensities–both in terms of $850\;\textrm{hPa}$ vorticity and mean sea level pressure–compared to reanalysis data (Seneviratne et al. 2021). They also suffer from biases in storm tracks and underestimate the frequency of cyclogenesis and explosive developments (Colle et al. 2013; Jiaxiang et al. 2020; Priestley et al. 2020; Zappa et al. 2013a). Moreover, there is significant spread among different climate models, with higher-resolution models generally performing better (Catto et al. 2019; Harvey et al. 2012; Jiaxiang et al. 2020). Priestley et al. (2020) and Harvey et al. (2020) highlighted notable improvements in the performance of models from the sixth phase of the Coupled Model Intercomparison Project (CMIP6) compared to earlier phases. These advancements are attributed to higher spatial resolution and improved model physics, enabling a more accurate representation of diabatic processes that can play a significant role in the formation of ETCs (Hirata et al. 2019; Wernli and Gray 2024). However, persistent biases remain, particularly in the representation of the most intense cyclones.

Regional climate models (RCMs) are valuable tools that complement global climate models (GCMs) by providing finer-scale detail for analyzing regional or local climate processes. Unlike GCMs, RCMs focus on limited regions, allowing for higher spatial resolution without excessive computational costs. This makes RCMs particularly suitable for studying small-scale and topography-related phenomena (Feser et al. 2011; Rummukainen 2010). Specifically, dynamical downscaling uses time-varying atmospheric variables (e.g., winds, temperature, water vapor, and surface pressure) and sea surface conditions (e.g., sea surface temperature and sea ice cover) obtained from the interpolation of coarse-resolution GCM-simulated data or reanalyses, to drive a higher resolution climate model over a limited region of the globe (Tapiador et al. 2020; Giorgi 2019). RCMs rely on lateral boundary conditions supplied by GCMs and occasionally utilize interior nudging, which integrates driving model information within the boundary zones to prevent deviations from the global model. The accurate treatment of these lateral boundary conditions is critical for reliable simulations, as errors in the boundary forcing can propagate into the regional domain (Xue et al. 2014). When properly implemented, dynamical downscaling should make the simulated weather more realistic and statistically closer to observations due to the development of small-scale features poorly developed or unresolved in GCMs. The primary challenge in applying RCMs is to leverage the advantages of higher resolution while minimizing the potential drawbacks associated with nesting within a limited domain. However, quantifying the added value of GCM-driven RCM simulations remains challenging (Di Luca et al. 2015). RCMs have dynamic downscaling capability only under specific conditions, including appropriate lateral boundary conditions, proper domain configuration, convective schemes, land surface parameterizations, initialization methods, and numerical schemes, as well as sufficiently large domains. Any significant shortcomings in these aspects can compromise an RCM’s dynamic downscaling ability (Xue et al. 2014). Nevertheless, it has been frequently shown that GCM-driven RCMs generally perform better at medium and small scales, and can improve some non-driven large-scale variables (Di Luca et al. 2016a; Seiler et al. 2018). They also provide added value in reducing the bias in the frequency of explosive cyclones along the Atlantic coast of North America simulated by GCMs (Seiler et al. 2018; Poan et al. 2018).

Therefore, the ranking of models is crucial when selecting a subset of GCMs for dynamical downscaling, as the quality of the lateral boundary conditions (i.e., the large-scale fields) they supply plays a critical role in the performance of the downscaling. In this study, we aim to evaluate CMIP6 models based on their ability to accurately represent the synoptic conditions associated with the most intense low-pressure systems, which are likely to result in precipitation and wind extremes in mid-latitudes. Additionally, we assess the added value of increased horizontal resolution provided by regional simulations compared to both the driving GCMs and other available GCMs. The methodology uses a novel Eulerian framework to identify the most extreme low-pressure systems at individual grid points based on the mean sea level pressure field (*p*) and the associated near-surface humidity (*q*). By considering both dynamical (i.e., *p*) and thermodynamical (i.e., *q*) fields, the proposed method provides a means to characterize favorable conditions for wind speed and precipitation extremes to occur (Field and Wood 2007; Rudeva and Gulev 2011). The Eulerian framework presented here decomposes the signature of extreme low pressure systems, at individual grid points, using three terms with a clear physical meaning. This enables a detailed analysis of model performance and the contribution of spatial resolution to the accuracy of both the thermodynamic and dynamic local atmospheric responses. While the focus of the study is on midlatitude regions and extratropical cyclones, the Eulerian algorithm will also capture tropical cyclones (Seneviratne et al. 2021) and their extratropical transition (Garin et al. 2025).

The paper is structured as follows. Section 2 presents a description of the data used, and Section 3 describes the Eulerian method and the linear approximation used to model the signature of the passage of low pressure systems as part of the methodology. Section 4 presents the results, including the validation of the linear approximation, an analysis of the parameters derived from this approximation, and the performance evaluation of the models. Lastly, discussion and concluding remarks are given in Section 5.

## Data

### Reanalysis data

Reanalysis products are commonly used to evaluate climate models and are often considered as a primary choice when studying ETCs, due to their quasi-continuous spatial and temporal representation of the atmospheric state (Chen et al. 2022; Chen and Luca 2025; Hodges et al. 2011; Priestley et al. 2020). The ERA5 reanalysis (Hersbach et al. 2020), developed by the European Centre for Medium-Range Weather Forecasts (ECMWF), is used as the reference against which the climate models are evaluated. ERA5 provides data at hourly intervals, with a TL639 spectral resolution, corresponding approximately to a $0.28^{\circ }$ $\times$ $0.28^{\circ }$ grid (about 31 km horizontal resolution at the equator). The variables used to capture both the dynamical and thermodynamical conditions at individual grid points during the passage of an ETC are the mean sea level pressure (*p*) and near-surface specific humidity (*q*). The data span a 35-year period, from 1980 to 2014. Both surface pressure and near-surface humidity are assimilated variables in ERA5 (Soci et al. 2024). Surface pressure observations are widely assimilated over both land and ocean, while near-surface humidity observations are assimilated primarily over land. Hence, their values in ERA5 are strongly constrained by observational data, and therefore are expected to reflect real-world conditions more closely than variables that are purely model-derived.

### CMIP6 GCM simulated data

Mean sea level pressure and near-surface specific humidity every 6 h from 27 Atmosphere-Ocean Global Climate Models (AOGCMS) from the Coupled Model Intercomparison Project Phase 6 (CMIP6, Eyring et al. 2016) are used in this study (Table 1). For each CMIP6 model, a single realization for the historical experiment for the period 1980-2014 is considered. The selection of CMIP6 models was based solely on the availability of the two variables over the period of interest.

### CRCM6 simulated data

We use the sixth version of the Canadian Regional Climate Model (CRCM6-GEM5) to perform multiple simulations over North America. CRCM6-GEM5 is based on version 5.1.1 of the Global Environmental Multiscale model (GEM5) which is the operational numerical weather prediction model at the Meteorological Service of Canada. The GEM5 model is developed by Environment and Climate Change Canada and is described in McTaggart-Cowan et al. (2019). Over lakes, the GEM5 model is coupled with the one-dimensional Fresh-water Lake model (FLake) (Martynov et al. 2012; Mironov et al. 2010). FLake represents lake surface temperatures and ice thickness based on a two-layer parametric model. The CRCM6-GEM5 model is described in Roberge et al. (2024); Whittaker et al. (2025) and Veilleux et al. (2025). CRCM6-GEM5 simulations cover the period from January 1980 to December 2014 and were performed using a $0.11^{\circ }$ rotated-pole grid over the CORDEX North American domain (Giorgi and Gutowski 2015). Two simulations were driven by the ERA5 reanalysis, one using only lateral boundary conditions (CRCM6-GEM5-UAA) and another using lateral boundary conditions and spectral nudging (CRCM6-GEM5-UAA-SN). Spectral nudging in this simulation is applied to horizontal winds and temperature above $850\;\textrm{hPa}$, targeting only large-scale waves with wavelengths greater than 200 km using a relaxation time scale of 8 h. Spectral nudging helps maintain strong consistency between the large-scale patterns of the simulation and those of ERA5. While the ERA5-driven simulation with spectral nudging includes nudging above $850\;\textrm{hPa}$, the GCM-driven simulations include nudging above 700 hPa. The choice of the lowest nudged level is inherently somewhat arbitrary and varies across previous studies (Wamahiu et al. 2025; Xu and Yang 2015; Takhsha et al. 2018), but we use $700\;\textrm{hPa}$ with GCM-driven simulations to moderately constrain de large-scale flow while preserving the RCM simulated conditions close to the surface. The remaining three simulations, CRCM6-GEM5-UBD, CRCM6-GEM5-UBE, and CRCM6-GEM5-UBF, were driven at the lateral boundaries by the EC-Earth3-Veg, the MPI-ESM1-2-HR, and the MIROC6 CMIP6 AOGCMs, respectively, with spectral nudging applied to horizontal winds above $700\;\textrm{hPa}$. In GCM-driven simulations, temperature is not nudged in order to avoid constraining the thermodynamic response of the regional model and to retain consistency with the large-scale climate signal provided by the driving GCM. These GCMs were selected based on data availability for downscaling, a preliminary bias assessment over North America, and their relatively high resolution compared to other available models. Table A1 provides more information on the configuration of the simulations used.

**Table 1:** Name, institution, ensemble member IDs, mean resolution in km and in deg° of each climate model used in this study and the resolution group to which they have has been assigned. Resolution groups correspond to classes of models with comparable horizontal grid spacing, allowing the added value of resolution to be assessed in Sect. 4.4. $\overline{\Delta x}$ corresponds to the average zonal and meridional grid spacing for each dataset ($\overline{\Delta x} = \sqrt{\Delta x \cdot \Delta y}$)

Model	Institution	Member IDs	$\overline{\Delta x}$ (km)	$\overline{\Delta x}$ (deg°)	Resolution
ACCESS-CM2	CSIRO-ARCCSS	r1i1p1f1	173.5	1.53	Low
ACCESS-ESM1-5	CSIRO	r1i1p1f1	173.5	1.53	Low
AWI-ESM-1-1-LR	AWI	r1i1p1f1	208	1.88	Low
BCC-CSM2-MR	BCC	r1i1p1f1	125	1.125	Middle
CanESM5	CCCma	r1i1p2f1	313	2.81	Low
CMCC-CM2-SR5	CMC	r1i1p1f1	120	1.075	High
CMCC-ESM2	CMC	r1i1p1f1	120	1.075	High
CNRM-CM6-1-HR	CNRM-CERFACS	r1i1p1f1	55	0.5	High
CNRM-CM6-1	CNRM-CERFACS	r1i1p1f1	156	1.4	Middle
CNRM-ESM2-1	CNRM-CERFACS	r1i1p1f2	156	1.4	Middle
CRCM6-GEM5-UAA	ESCER-ECCC	$\backslash$	12	0.11	$\backslash$
CRCM6-GEM5-UAA-SN	ESCER-ECCC	$\backslash$	12	0.11	$\backslash$
CRCM6-GEM5-UBD	ESCER-ECCC	$\backslash$	12	0.11	RCM
CRCM6-GEM5-UBE	ESCER-ECCC	$\backslash$	12	0.11	RCM
CRCM6-GEM5-UBF	ESCER-ECCC	$\backslash$	12	0.11	RCM
EC-Earth3-AerChem	ECEC	r1i1p1f1	80	0.7	High
EC-Earth3-Veg-LR	ECEC	r1i1p1f1	126	1.13	Middle
EC-Earth3-Veg	ECEC	r1i1p1f1	80	0.7	High
EC-Earth3	ECEC	r1i1p1f1	80	0.7	High
GFDL-CM4	NOAA-GFDL	r1i1p1f1	100	1	High
GFDL-ESM4	NOAA-GFDL	r1i1p1f1	100	1	High
GISS-E2-1-G	NASA-GISS	r1i1p1f1	250	2.24	Low
KIOST-ESM	KIOST	r1i1p1f1	208	1.875	Low
MIROC-ES2L	MIROC	r1i1p1f1	313	2.81	Low
MIROC6	MIROC	r1i1p1f1	156	1.4	Middle
MPI-ESM-1-2-HAM	MPI-M	r1i1p1f1	209	1.875	Low
MPI-ESM1-2-HR	MPI-M	r1i1p1f1	104	0.9375	High
MPI-ESM1-2-LR	MPI-M	r1i1p1f1	209	1.875	Low
MRI-ESM2-0	MRI	r1i1p1f1	125	1.125	Middle
NorESM2-MM	NCC	r1i1p1f1	122	1.09	Middle
SAM0-UNICON	SNU	r1i1p1f1	122	1.09	Middle
TaiESM1	AS-RCEC	r1i1p1f1	122	1.095	Middle

## Methods

### Regridding data

To compare data with different horizontal and temporal resolutions, we postprocess all data (i.e., ERA5, CRCM6-GEM5 and CMIP6 model data) to ensure that they provide information at similar spatiotemporal scales (Di Luca et al. 2016b; Prein et al. 2016). We perform the comparison between simulations and reanalysis using a common grid mesh of $1.5^{\circ }$ in latitude and longitude. All reanalysis and model simulations are regridded onto this common $1.5^{\circ }$ grid using bilinear interpolation methods. The remapping ensures that the information at spatial scales finer than $1.5^{\circ }$ is filtered out although such a methodology does not alter the influence that fine scales could have had on the large scales. Similarly, the comparison is performed using instantaneous fields available every 6 h, which corresponds to the highest temporal resolution shared by all datasets. In the rest of the paper, the ensemble-mean is constructed using all AOGCM and RCM simulations, except for the two RCM simulations driven by ERA5, in order to avoid any dependency bias when evaluating performance against ERA5 data.

### Finding Eulerian storms

**Fig. 1:**
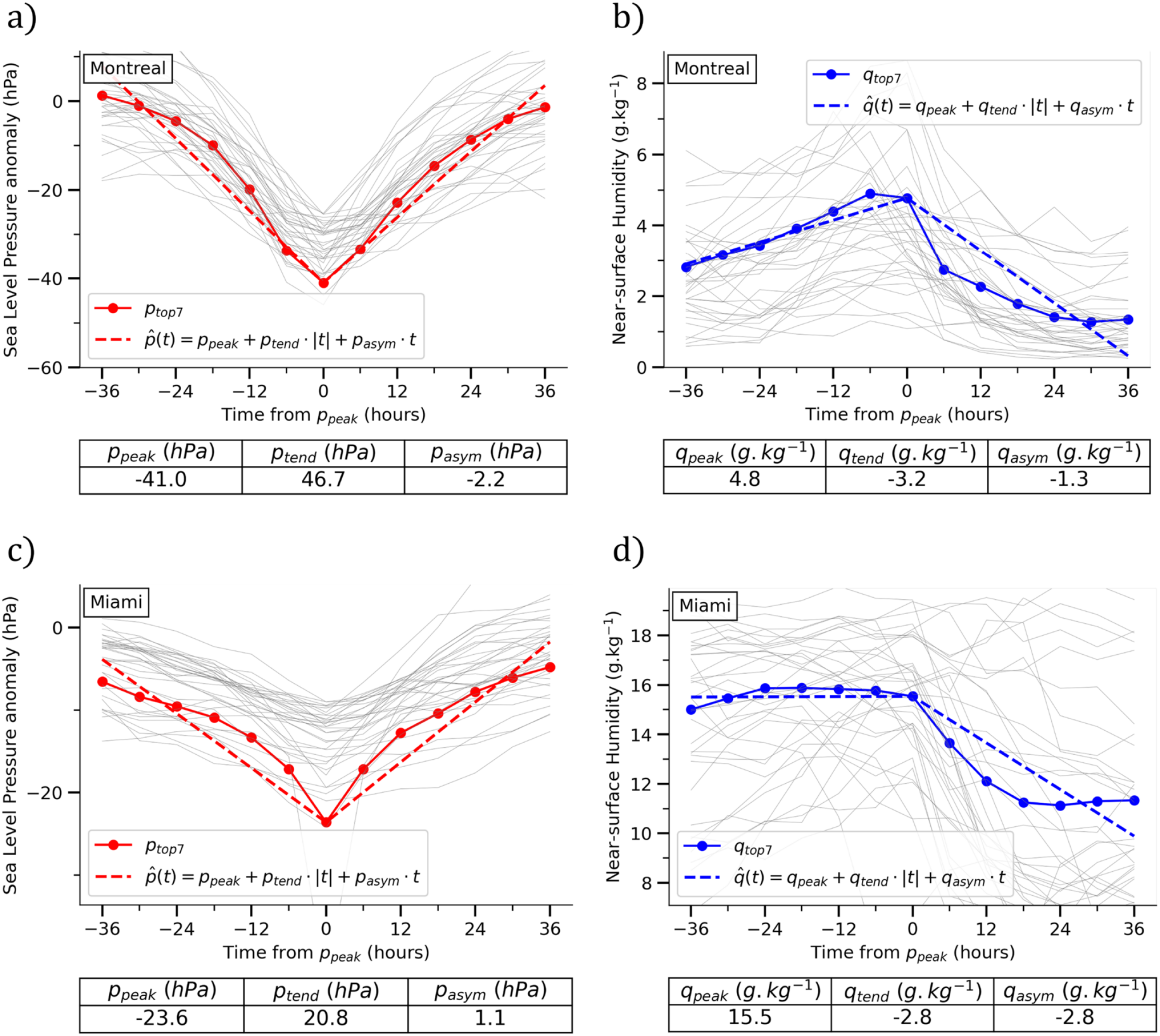
Mean sea level pressure anomalies (left panels) and associated near-surface humidity (right panels) 36 h before and after the passage of the strongest storm in each year between 1980 and 2014 (grey lines) for the grid points closest to Montreal (top panels) and Miami (bottom panels). The solid lines in all panels are the average using the strongest 7 storms and the dashed line results obtained using a linear approximation with 3 parameters. All time series are from the ERA5 reanalysis. The tables below each panel show the value of each coefficient from Eq. 1

Storms are identified at each grid point by locating the minima in the 6-hourly time series of mean sea level pressure anomalies. These 6-hourly anomalies are computed from the climatological mean sea level pressure values of the Julian day, defined as the average over the 35-year period from 1980 to 2014 for each calendar day. The idea of removing the mean annual cycle of mean sea level pressure is to remove the influence of seasonal variations in the storm’s selection. For simplicity, the anomalous mean sea level pressure is denoted by *p* and the minimum anomaly for each year *y* is denoted by $p_{min}^y$. For each identified minimum value $p_{min}^y$, we extract 6-hourly time series of *p* and *q* for the 3-day period around the date of $p_{min}^y$ (i.e., 36 h before and after the occurrence of the minimum pressure anomaly value). To illustrate the methodology, Fig. 1 shows an example of extracted 3-day time series for the grid points closest to Montreal (top panels) and Miami (bottom panels). Grey lines show ERA5 3-day time series of *p* (left panels) and *q* (right panels) for yearly identified storms between 1980 and 2014. A total of 35 lines are shown, corresponding to the 35 yearly low-pressure systems with the highest pressure anomaly magnitude at the grid point. In addition, solid red and blue lines show the time series resulting from the average of the 7 storms with the lowest value of $p_{min}^y$, for *p* (right panels) and *q* (left panels) respectively. Throughout this study, the analysis is systematically conducted on these 7 strongest storms, i.e., those with the lowest values of $p_{min}^y$ at each grid point, rather than on the full set of 35 yearly systems. As expected, in both locations, the pressure anomaly changes substantially during the passing of the storm with pressure changes of about $40\;\textrm{hPa}$ and $20\;\textrm{hPa}$ in Montreal and Miami respectively. The associated humidity also shows important changes, with a slight increase or no change before the occurrence of $p_{min}^y$ and a strong decrease after.

Figure 1 also shows a fit using the overlap of two linear approximations: a linear approximation that fits the time series 36 h before the minima occurs ($\widehat{y_b}(t) = a_b \cdot t + c$) and a linear approximation that fits the time series 36 h after the minima ($\widehat{y_a}(t) = a_a \cdot t + c$). For all times $t = -36h + i \cdot 6$ with $i \in [0,12]$, this fit can be expressed for *p* as:

1 $$\begin{aligned} \widehat{p}(t) = p_{peak} + p_{tend} \cdot |t| + p_{asym} \cdot t \end{aligned}$$

with $p_{\text {asym}} = \frac{a_b + a_a}{2}$ and $p_{tend} = \frac{a_a - a_b}{2}$. The linear fit can be applied to both *p* and *q* time series and, in both cases, the interpretation of the three coefficients in Eq. 1 is as follows:

- *Peak* value: the parameter *peak* indicates the value of *p* or *q* at the time of the minimum pressure anomaly $p_{min}^y$. That is, $p_{peak} = p_{min}^{top7}$ for *p*. The value of *q* at the time of the minimum pressure anomaly is $q_{peak} = q^{top7}(t=0) \ge 0$. It is important to note that $q_{peak}$ is not necessarily equal to $q_{max}^{top7}$, since our primary objective is to identify low-pressure systems based on the magnitude of their pressure anomaly and to analyze the associated near-surface humidity.
- Mean *tendency*: the parameter *tendency* $\frac{a_a - a_b}{2}$ provides an estimation of the difference of the rate of change of *p* or *q* after and before the time of the minimum pressure anomaly. For the variable *p*, the slopes before and after the minimum pressure anomaly are usually negative and positive, respectively, and thus $p_{tend} \ge 0$. It provides an estimate of the mean local tendency rate observed at a given grid point, with $\left[ p_\textrm{tend}\right] = \mathrm {hPa\,h^{-1}}$. For the variable *q*, $q_{tend}$ provides an estimate of the mean rate of change in near-surface specific humidity 36 h before and after the pressure anomaly minimum, with $\left[ q_\textrm{tend}\right] = \mathrm {g\,kg^{-1}\,h^{-1}}$. If *q* corresponds to a local maximum at the time of the pressure anomaly minimum, then $q_{tend} \le 0$. In the following sections of the paper, the values of $p_{tend}$ and $q_{tend}$ are multiplied by 36 to be expressed in $\textrm{hPa}$ and $\mathrm {g\,kg^{-1}}$, respectively.
- Tendency *asymmetry*: the parameter *asymmetry* $\frac{a_b + a_a}{2}$ provides an estimation of the average of the rate of change after and before the time of the minimum pressure anomaly. For the variable *p*, the slopes before and after the cyclone’s passage have opposite signs, and $p_{asym}$ provides a measure of the asymmetry of the cyclone signature, with $\left[ p_\textrm{asym}\right] = \mathrm {hPa\,h^{-1}}$. A symmetric signature (e.g., a perfect V) would lead to $p_{asym} = 0$, and the larger the absolute value of $p_{asym}$, the more asymmetric the signature of the cyclone would be. Positive (negative) values of $p_{asym}$ indicate that the rate of pressure drop before the minimum is lower (higher) than the rate of pressure rise after the minimum. A similar reasoning can be made for $q_{asym}$, but with positive (negative) values indicating a rate of near-surface humidity increase larger (lower) than that of decrease, with $\left[ q_\textrm{asym}\right] = \mathrm {g\,kg^{-1}\,h^{-1}}$. The values of $p_{asym}$ and $q_{asym}$ are also multiplied by 36 to be expressed respectively in $\textrm{hPa}$ and $\mathrm {g\,kg^{-1}}$ in the following sections of the paper.

The three parameters defined here are derived from an Eulerian perspective but they are related to parameters traditionally used when identifying ETCs and TCs following a Lagrangian perspective. The Eulerian pressure minimum parameter relates to the Lagrangian center of the storm (Di Luca et al. 2016b; Neu et al. 2013; Schneidereit et al. 2010; Simmonds and Keay 2000), but unless the storm center passes exactly through the grid point of interest, then the Eulerian pressure minimum will be higher than the Lagrangian one. The Eulerian mean *tendency* parameter is related to the Lagrangian depth, that measures the difference between the storms’ central pressure and the surrounding pressure at a distance equal to the radius of the cyclone (e.g., Schneidereit et al. 2010; Simmonds and Keay 2000). However, the Eulerian mean *tendency* depends not only on the Lagrangian depth, but also on the propagation speed (Neu et al. 2013) and the size of the storm. For example, assuming that the pressure field around the storm remains unchanged during a certain time period, the pressure tendency in a grid point will be greater if the storm moves faster than if it moves slower. Similarly, for a cyclone with a given central pressure minimum and a propagation speed, the cyclone with the smaller radius will generally show a larger pressure tendency. The *asymmetry* term is also the result of a combination of factors that cannot be unambiguously disentangled from an Eulerian perspective alone. The storm’s propagation speed, its trajectory relative to a given grid point, or local effects, such as diabatic processes or strong baroclinicity, can all influence the local deepening or filling rate of *p* or *q*, and thus the symmetry of the signal associated with the local passage of low-pressure systems.

### Evaluation metrics

Storms are selected for all grid points over a region that includes North America for all years in the period 1980-2014 for the ERA5 reanalysis, the 27 CMIP6 model simulations and the 5 CRCM6-GEM5 regional model simulations. After selecting and averaging the 7 storms with the lowest $p_{peak}$ in each grid point, we obtain a time series of *p* and *q* for each dataset (reanalysis and models) at each grid point. At any given grid point, the mean absolute error (*mae*) of the pressure anomaly (*p*) or the associated humidity (*q*) can be calculated as:

2 $$\begin{aligned} y_{mae} = \frac{1}{N} \sum \limits _{t_i=-36}^{36} \left| y_m(t_i) - y_{ERA5}(t_i) \right| , \ t_i \in \{-36 + 6i \mid i \in [1,13] \} \end{aligned}$$

where the subindex *m* denotes a given model and ERA5 denotes the reanalysis. The errors are computed as the average of absolute errors over the 3-day period surrounding the minimum pressure anomaly, with $N=13$, as there are 13 time steps of 6 h within this period. Using the linear approximation from Eq. 1, the *mae* from Eq. 2 can be approximated for *p* using only three parameters as follows:

3 $$ \begin{aligned} y_{{\widehat{{mae}}}} = & \frac{1}{N}\sum\limits_{{t_{i} = - 36}}^{{36}} {\left| {\hat{y}_{m} (t_{i} ) - \hat{y}_{{ERA5}} (t_{i} )} \right|} \\ = & \frac{1}{N}\sum\limits_{{t_{i} = - 36}}^{{36}} | \left( {p_{{peak_{m} }} - p_{{peak_{{ERA5}} }} } \right) \\ & + \left( {p_{{tend_{m} }} - p_{{tend_{{ERA5}} }} } \right)|t_{i} | \\ & + \left( {p_{{asym_{m} }} - p_{{asym_{{ERA5}} }} } \right)t_{i} | \\ = & \frac{1}{N}\sum\limits_{{t_{i} = - 36}}^{{36}} | p_{{peak}}^{{bias}} + p_{{tend}}^{{bias}} |t_{i} | + p_{{asym}}^{{bias}} t_{i} | \\ \end{aligned} $$

Note that the same reasoning can be done for *q*. Defined this way, the $\widehat{mae}$ error only depends on three parameters that have a clear physical meaning and that measure the contribution to the total error by errors in the *peak*, the *tendency* and the *asymmetry*. Equation 3 suggests that some models may achieve a low *mae* by compensating for errors across individual parameters–for instance, an underestimated *peak* offset by an overestimated mean *tendency*. To address this issue and identify climate models that produce low errors for the right reasons or prevent misleading information from error compensation, we define a new model error for *p* (also applicable to *q*) as follows:

4 $$ \begin{aligned} y_{{\widehat{{aae}}}} = & \frac{1}{N}\sum\limits_{{t_{i} = - 36}}^{{36}} {\left| {\left( {p_{{peak_{m} }} - p_{{peak_{{ERA5}} }} } \right)} \right|} \\ & + \left| {\left( {p_{{tend_{m} }} - p_{{tend_{{ERA5}} }} } \right)t_{i} } \right| \\ & + \left| {\left( {p_{{asym_{m} }} - p_{{asym_{{ERA5}} }} } \right)t_{i} } \right| \\ = & p_{{peak}}^{{abs}} + p_{{tend}}^{{abs}} + p_{{asym}}^{{abs}} \\ \end{aligned} $$

This evaluation criteria is denoted as the additive absolute error and models will show low values of $\widehat{aae}$ only if they are able to well represent each individual term, that is, if their time series most closely match those of ERA5. By extension, any limitations of ERA5 will not be captured by the error metrics, since a model reproducing an ERA5 bias would appear to perform well; the metrics thus implicitly assume ERA5 to be a reliable reference. Throughout the paper, we refer to discrepancies between models and ERA5 as “errors” for convenience, although they should more rigorously be interpreted as differences relative to a reference that is itself an imperfect representation of the true atmospheric state and carries inherent uncertainties. For instance, Wu et al. (2024) highlighted ERA5’s deficiencies in characterizing pre-storm conditions compared to radiosonde observations across China. Similarly, Dulac et al. (2024) reported that ERA5 tends to underestimate the minimum sea-level pressure associated with tropical cyclones. ERA5 has also been shown to exhibit biases in convective environments when compared with radiosonde observations (Taszarek et al. 2021a, b). Other studies have also pointed out ERA5’s limitations in representing surface pressure and relative humidity compared to other reanalyses (Kara and Elbir 2024; Xu et al. 2025) or its performance issues over complex terrain (Chen et al. 2024; Minola et al. 2020). Similar approaches to define error metrics aiming at avoiding the compensation of errors can be found in Di Luca et al. (2020) and Luca et al. (2020).

## Results

### Evaluation of the linear approximation of *p* and *q*

**Fig. 2:**
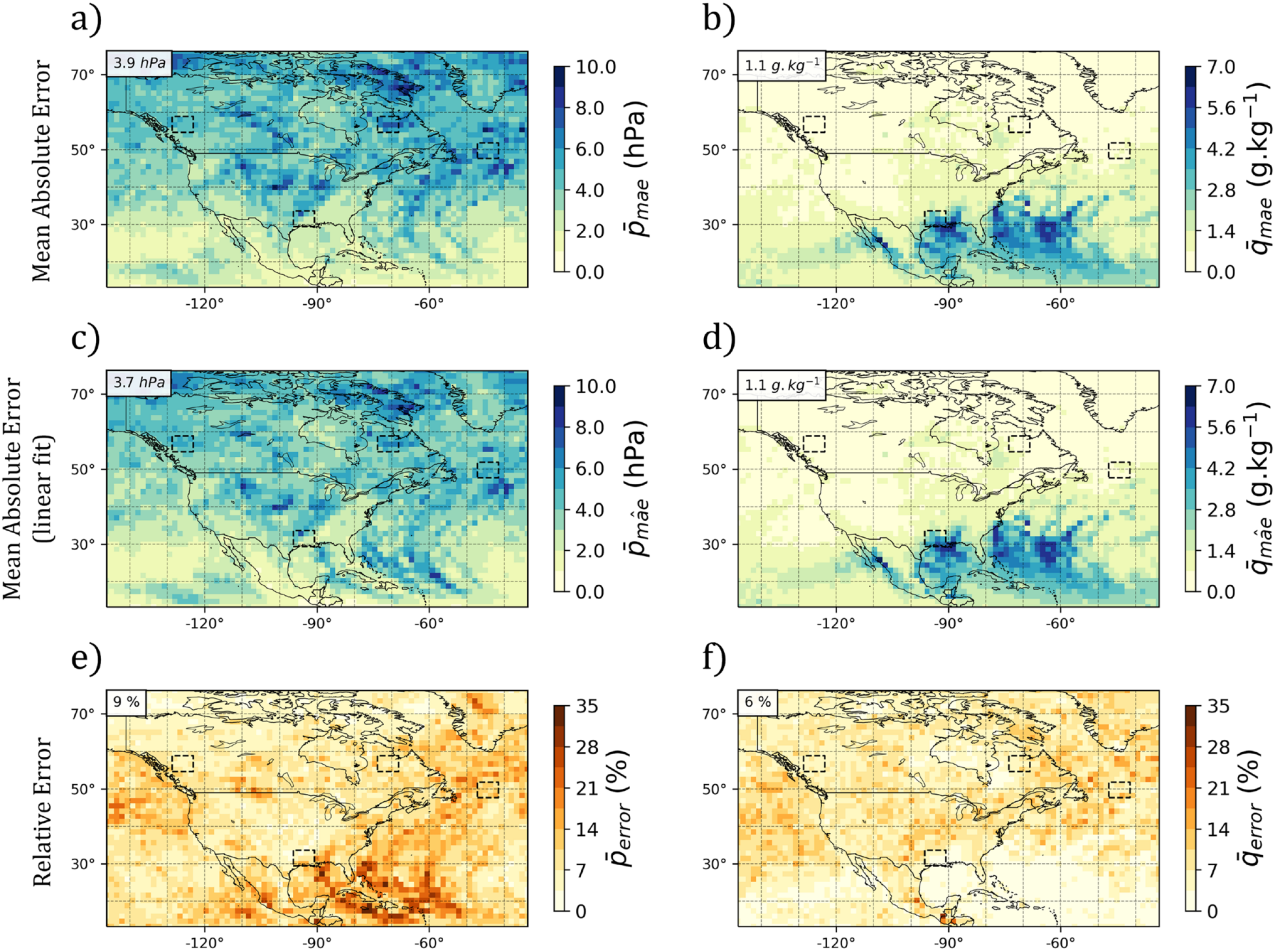
Model ensemble-mean of the mean absolute error (*mae*) (top panels), calculated with the linear fit ($\hat{mae}$) (middle panels) and the relative error between both (bottom panels) for pressure anomalies (left panels) and associated near-surface humidity (right panels). The dashed squares indicate regions analyzed in Section 4.3

Figure 2a depicts the spatial distribution of the model ensemble-mean of the mean absolute error of pressure anomalies ($\bar{p}_{mae}$) across North America (see Eq. 2). The mean absolute error ranges from $0\;\text {to}\;10\;\textrm{hPa}$ over the 3-day time series, with the lowest values observed in the tropical regions. Conversely, the highest errors are found in the inland areas of the United States and the northern regions of Canada, particularly in the western North Atlantic. The ensemble mean of $\bar{p}_{mae}$ over the whole domain is $3.9\;\textrm{hPa}$. Some tropical cyclone tracks appear in the model errors along the east coast of the continent, likely due to the challenges climate models face in accurately simulating tropical cyclones in this area (Camargo 2013; Walsh et al. 2016).

Figure 2c shows the model ensemble-mean error obtained using the linear approximation presented in Eq. 3. The domain-average mean error of pressure anomalies ($\bar{p}_{\widehat{mae}}$) is $3.7\;\textrm{hPa}$, and the spatial distribution closely resembles that shown in Fig. 2a with the exception of some areas in the tropical Atlantic, where errors are larger than in Fig. 2a. The relative error between the total model error and its linear approximation (in %) is shown in Fig. 2e. In the oceanic tropical regions, the linear approximation of the simulated 3-day time series of pressure anomalies does not accurately capture the observed patterns and shows a relative error of about $30\;\%$, while the domain average is $9\;\%$. Therefore, we suggest that the linear approximation is more suitable for mid-latitude regions where errors are generally within 10$\%$, while it is less suitable over tropical regions where the cyclone signatures exhibit non-linear characteristics from an Eulerian perspective.

As for the near-surface humidity, the mean absolute error of the ensemble-mean of the near-surface humidity ($\bar{q}_{mae}$) ranges from $0\;\text {to}\;7\,\mathrm {g\,kg^{-1}}$ over the 3-day time series, with a mean value of $1.1\,\mathrm {g\,kg^{-1}}$ across North America (Fig. 2b). The highest errors are observed in the Atlantic Ocean below latitude $40^{\circ }$, with a notable concentration in the Gulf of Mexico and north of the Carribean Sea. Additionally, some inland grid points near coastal areas also exhibit significant errors, particularly along the west coast of Mexico and in Louisiana and neighboring states in the US. In other regions, $\bar{q}_{mae}$ rarely exceeds $3\,\mathrm {g\,kg^{-1}}$, which is expected giving the lower value of *q* outside of the tropics. The mean absolute error for the linear approximation of near-surface humidity ($\bar{q}_{\widehat{mae}}$) exhibits a similar geographical pattern to that of $\bar{q}_{mae}$, with a domain-wide average also equal to $1.1\,\mathrm {g\,kg^{-1}}$ (Fig. 2d). However, certain oceanic grid points above $45^{\circ }$N exhibit high errors, reaching up to $20\;\%$, as do areas in the Great Plains and Central America (Fig. 2f). The domain average of the absolute relative error is $6\;\%$, indicating that the linear approximation provides a reasonably good characterization of the near-surface humidity errors.

### Eulerian cyclones in ERA5 and simulations

**Fig. 3:**
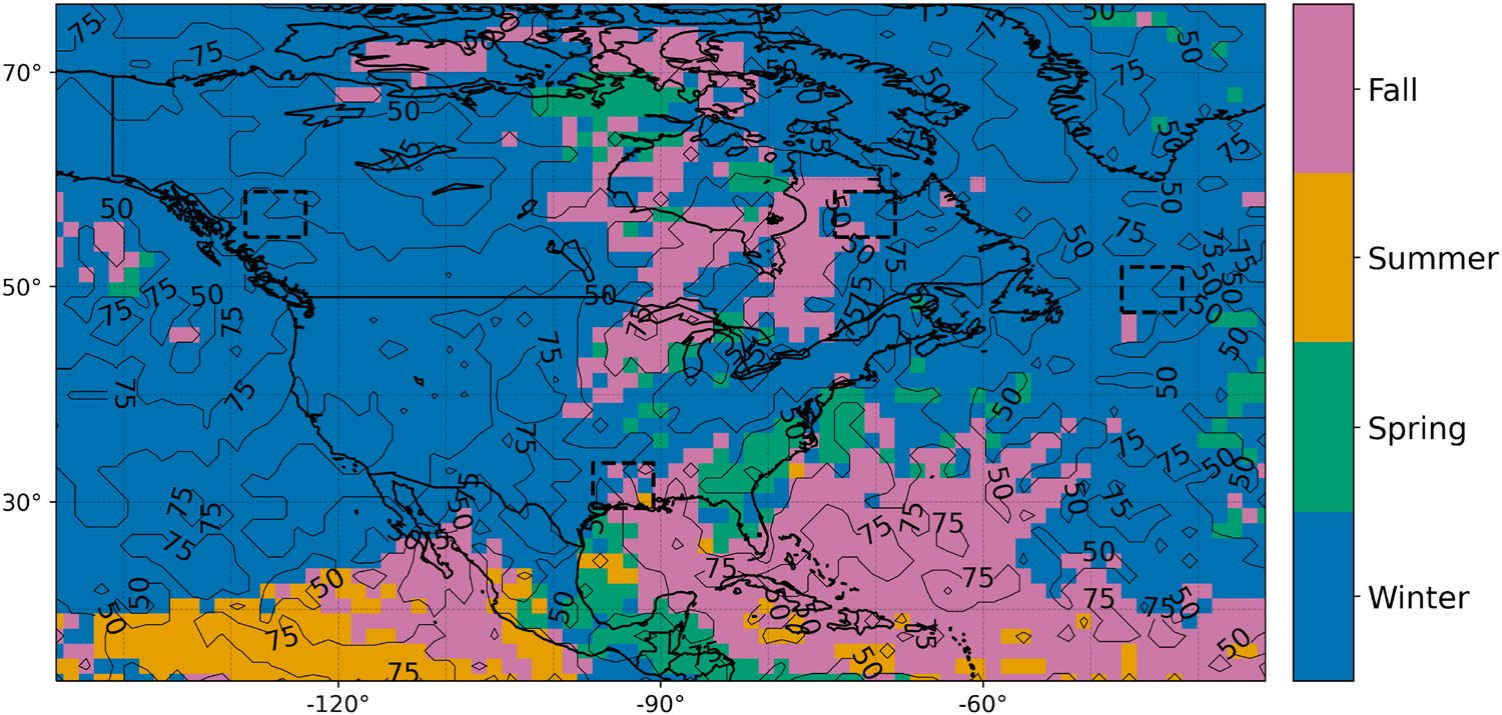
Dominant season among the 7 strongest storms identified by the Eulerian algorithm in ERA5 between 1980 and 2014. The black contour lines represent the proportion (in $\%$) of the 7 storms occurring during the dominant season. The dashed squares indicate regions analyzed in Section 4.3

Our Eulerian detection method is based on identifying the strongest yearly pressure anomaly at each grid point. As mentioned in the Methods section, 6-hourly anomalies are calculated relative to the multiyear calendar-day mean to remove the influence of seasonal-mean variations in the pressure field. Consequently, the strongest storms can occur at any time of the year. Figure 3 shows the season in which events among the seven strongest storms most frequently occur, at each grid point. Most grid points located in the mid-latitudes exhibit the strongest pressure anomalies occurrences during the winter season, when extratropical cyclones are stronger and more frequent, consistent with the findings of Plante et al. (2015), who quantified Lagrangian-tracked ETC intensity using relative vorticity at $850\;\textrm{hPa}$. In contrast, the tropics reveal a higher frequency of fall and summer cyclones, indicating the likely detection of tropical cyclones. The regions surrounding the Great Lakes and Hudson Bay exhibit more variations, with the fall season being often the most frequent, as in Plante et al. (2015). The results suggest that our Eulerian detection method can capture a diverse range of low-pressure systems across North America, consistent with expected patterns and characteristics.

**Fig. 4:**
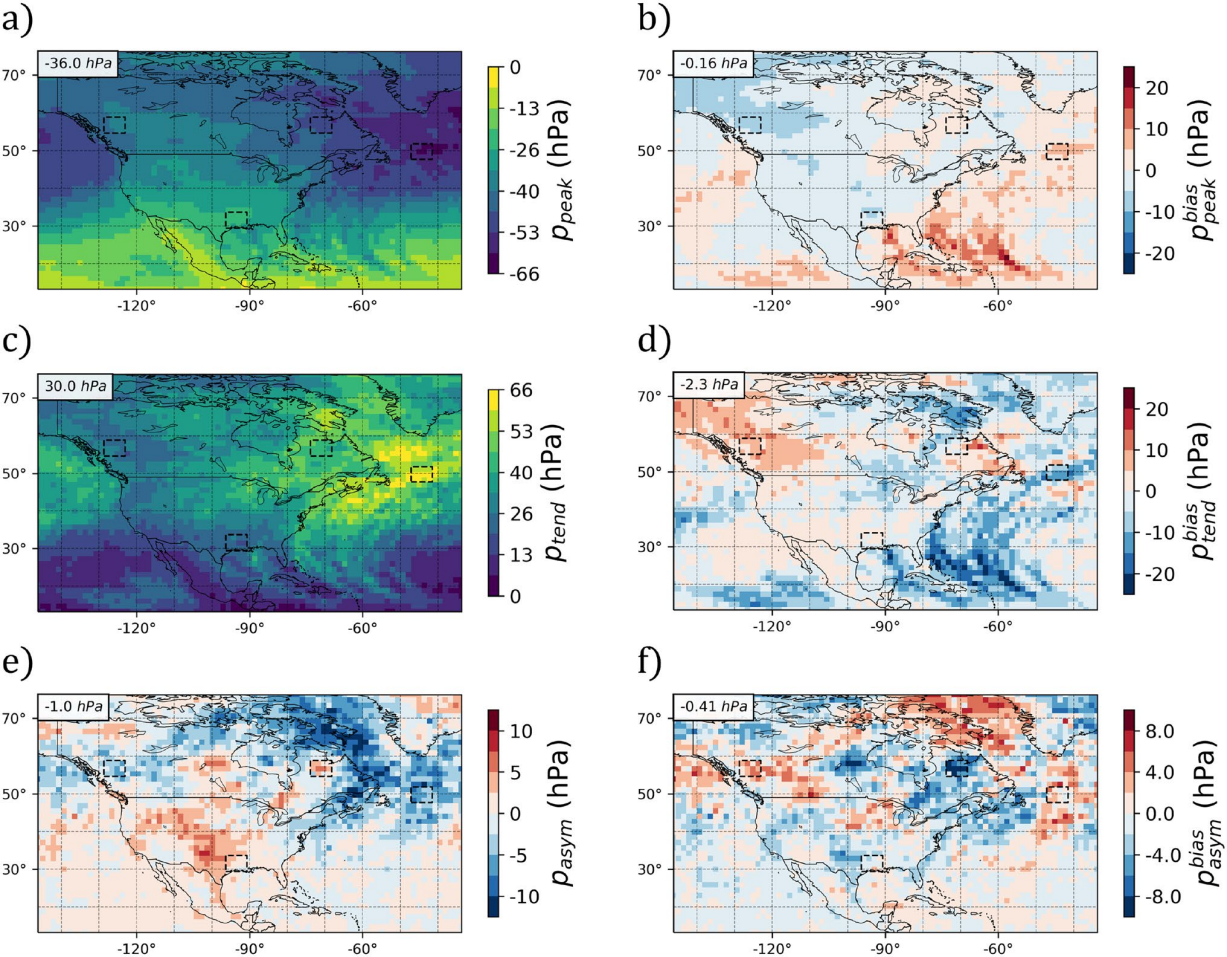
Pressure anomaly (*p*) *peak* (top panels), *tendency* (middle panels) and *asymmetry* (bottom panels) parameters as obtained from the ERA5 reanalysis (left panels) and the corresponding model ensemble-mean bias (ensemble-mean - ERA5) (right panels). The dashed squares indicate regions analyzed in Section 4.3

Left panels in Fig. 4 show the spatial distribution of the three linear-regression parameters (*peak*, *tendency*, and *asymmetry*) derived from the ERA5 mean sea level pressure. The parameter $p_{peak}$ represents the pressure anomaly at the time of the minimum and verifies $p_{peak}<=0$. Particularly in oceanic regions, $p_{peak}$ shows a strong north–south gradient; the magnitude of the anomalies is weak in the tropics and much larger in mid and high latitudes (Fig. 4a). Although tropical cyclones around North America can reach some of the lowest central pressures observed in the region, their associated pressure anomalies do not appear particularly strong in Fig. 4a. This is likely due to the regridding onto the $1.5^{\circ }$ common grid, which does not adequately resolve the spatial structure of tropical cyclones. The present analysis is therefore better suited for ETCs, even though tropical systems are still detected. The North Atlantic exhibits the largest magnitude of pressure anomalies, reaching up to $-60\;\textrm{hPa}$, while the domain average of $p_{peak}$ is $-36\;\textrm{hPa}$. The magnitude of the pressure anomaly is generally stronger over ocean than over the land. The large values observed over northeastern North America correspond to the region where the most intense and most frequent ETCs are observed during the winter months (Poan et al. 2018). Figure 4b shows the bias of the model ensemble-mean ($\bar{p}_{peak}^{bias}$), computed by averaging the results from 30 model simulations (the two CRCM6 simulations driven by ERA5 are excluded). $\bar{p}_{peak}^{bias}$ shows that overall, the models tend to underestimate pressure anomalies over oceanic regions and overestimate them over land regions (except in a few areas in eastern Canada.). Particularly in Northwest North America, there is a large area that exhibits an ensemble-mean bias lower than $-5\;\textrm{hPa}$. Figure 4b also clearly shows the tracks of Atlantic tropical cyclones, with an underestimation of the strongest pressure anomalies of approximately $15\;\textrm{hPa}$ (Fig. 4b).

The parameter $p_{tend}$ quantifies the mean temporal rate of change in atmospheric pressure at a specific grid point during the 72-h passage of a storm, encompassing both the pressure drop phase as the storm arrives and the subsequent recovery phase. The spatial distribution of $p_{tend}$ (Fig. 4c) reveals a strong similarity with the magnitude of the pressure anomalies $p_{peak}$, which is expected given the physical connection between these two terms. In the North Atlantic, where pressure anomalies are strongest, the rate of pressure change is also highest, exceeding $1.65\;\mathrm {hPa\,h^{-1}}$ (equivalent of $60\;\textrm{hPa}$ on the map), surpassing the conventional threshold for explosive development ($1\;\textrm{hPa}$ per hour). Furthermore, $\bar{p}_{peak}^{bias}$ and $\bar{p}_{tend}^{bias}$ also show an opposite pattern across the domain (Fig. 4b and 4d), highlighting the compensatory relationship between the *peak* pressure anomaly and the rate of change in pressure of the low-pressure systems affecting local grid points. We note that caution must be exercised in interpreting pressure errors over the tropical Atlantic due to the poorer performance of the linear fit (Fig. 2e).

The parameter $p_{asym}$ quantifies the asymmetry in the rate of atmospheric pressure change before and after the time at which the minimum pressure is observed at a given grid point. The ERA5 *asymmetry* parameter reveals regions characterized by pronounced asymmetry in the pressure signature associated with storm passage (Fig. 4e). In particular, along the eastern coasts of North America and northeastern Canada, storms tend to exhibit a more rapid pressure deepening prior to the peak anomaly than the subsequent recovery phase. These regions correspond to strongly baroclinic environments during the cold season, with frequent cold-air advection from continental interiors toward warmer oceanic regions. The East Coast is especially prone to cyclone redevelopment or rapid intensification owing to enhanced low-level baroclinic gradients and the presence of diabatic potential vorticity generated by latent heat release over the ocean (Lackmann 2011). As a result, these coastal systems, often referred to as “Nor’easters” (Hirsch et al. 2001), are both frequent and potentially explosive. Consistent with this, the East Coast and northeastern Canada correspond to regions exhibiting the largest pressure anomalies (Fig. 4a) and the highest wintertime ETC intensities (Poan et al. 2018). Climate models struggle to accurately reproduce this asymmetric behavior, exhibiting a spatial gradient in bias ranging from positive over Baffin Island and Baffin Bay to negative over Quebec and the East Coast (Fig. 4f). Overall, the model ensemble mean displays systematic biases in regions characterized by strongly asymmetric storm evolution. In contrast, storms affecting the interior United States, particularly over the Great Plains in the lee of the Rockies, tend to exhibit a faster recovery phase relative to the deepening phase, a behavior that models also struggle to reproduce (Fig. 4f). Lastly, regions frequently affected by tropical cyclones tend to exhibit a relatively symmetric signature.

**Fig. 5:**
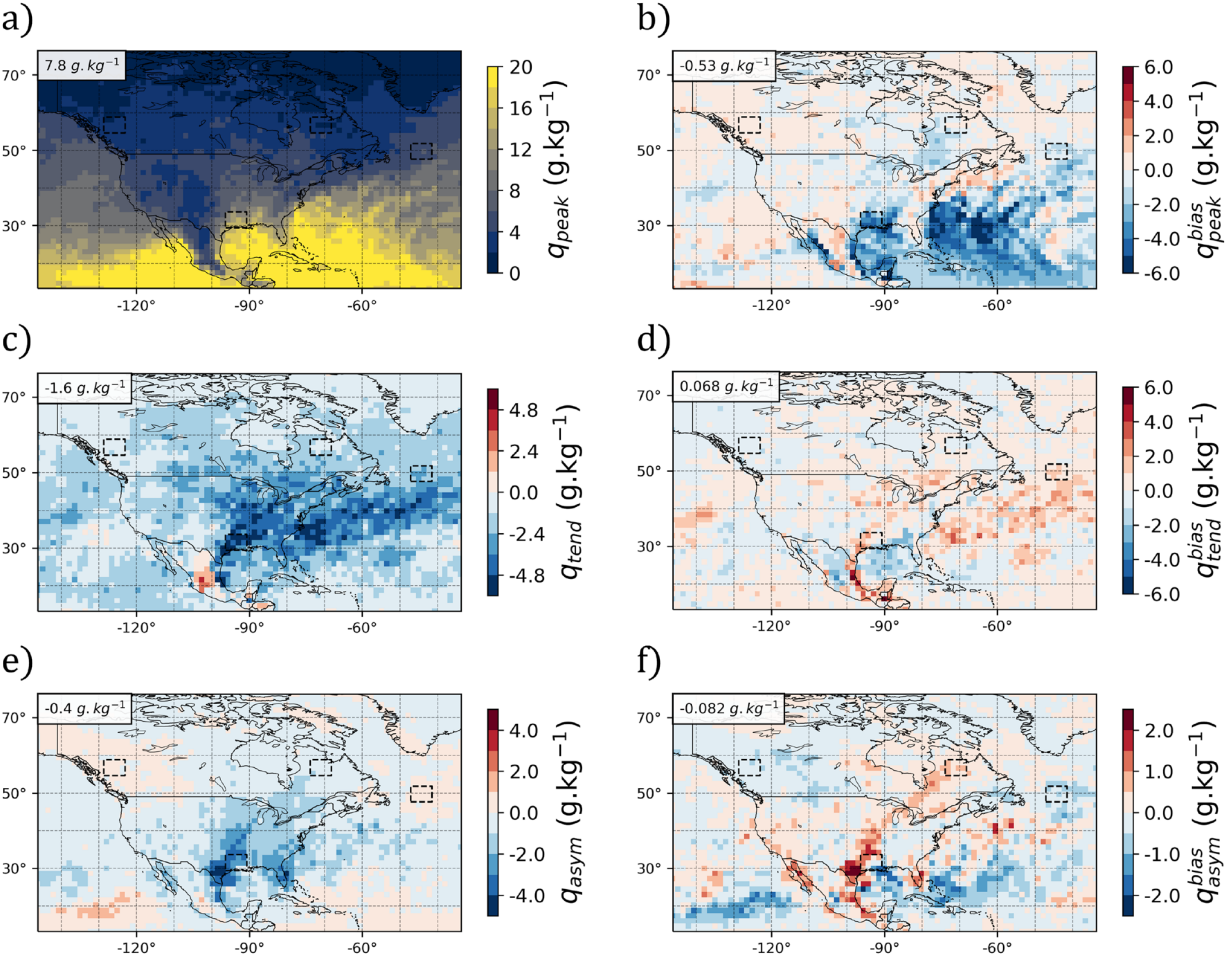
As in Fig. 4 but for near-surface humidity (*q*)

The ERA5 near-surface humidity at the time of the *peak* pressure anomaly ($q_{peak}$) exhibits a distinct north–south gradient, with values reaching up to $20\,\mathrm {g\,kg^{-1}}$ in tropical regions and below $5\,\mathrm {g\,kg^{-1}}$ in high-latitude regions (Fig. 5a). The bias of the model ensemble-mean, $\bar{q}_{peak}^{bias}$ (Fig. 5b), reveals significant underestimation where sea surface temperatures (SST) are high, in the North Atlantic below $40^{\circ }$N and along the Gulf of Mexico coastlines, consistent with the pattern of $\bar{q}_{mae}$ shown in Fig. 2b. The largest errors are observed in southern regions characterized by high $q_{peak}$ values. Although the bias is very small in the North Pacific, SST are lower than at the same latitudes in the North Atlantic. There are localized patches of slight positive bias over western Canada, Mexico, or offshore north of the East Coast, but the overall bias over the entire North American domain is negative, with a mean magnitude of $-0.53\,\mathrm {g\,kg^{-1}}$. ERA5 is known to underestimate the northward extent of moisture along the East Coast near the Gulf Stream (Taszarek et al. 2021b), which could explain the positive bias found in this region.

A negative value of the mean rate of change in near-surface humidity during the passage of a storm $q_{tend}$ indicates an overall increase in near-surface humidity as the storm approaches, followed by a subsequent decrease. This behavior is consistent with the expected response of a storm, which brings humidity locally, mainly before the passing of the cyclone (see Fig. 1). This pattern is observed over most of the domain in the ERA5 reanalysis (Fig. 5c), particularly over the southeastern United States and the North Atlantic, where the temporal rate of change of near-surface humidity during the storm passage is less than $-0.10\;\mathrm {g\,kg^{-1}\,h^{-1}}$ (or $-3.6\,\mathrm {g\,kg^{-1}}$ on the map). However, in parts of Mexico, Central America, and the North Pacific, a positive value of $q_{tend}$ is observed, suggesting a probable decrease in near-surface humidity before the passage of the storm. The model ensemble-mean $\bar{q}_{tend}$ exhibits a strong positive bias compared to ERA5 in the mid-latitudes of the North Atlantic, with bias values nearly opposite in sign to those of ERA5 (Fig. 5d). This suggests a weaker humidity change before and after the storm passage, potentially linked to warm or cold SST biases over the North Atlantic in most GCMs, as previously noted by Poan et al. (2018) and Zhang et al. (2023). Interestingly, the Gulf of Mexico shows negative errors, indicating a higher *tendency* rate in the models, despite the underestimation of *peak* humidity in this region. Grid points with significant bias are also found in the eastern part of North America, Mexico, Central America, and some areas of the North Pacific. However, the domain average of $\bar{q}_{tend}^{bias}$ is low, at $0.068\;\mathrm {g\,kg^{-1}\,h^{-1}}$ (i.e., $-2.45\,\mathrm {g\,kg^{-1}}$).

The parameter $q_{asym}$ in ERA5 observations exhibits a pronounced negative pattern over the eastern United States and parts of the North Atlantic, with stronger values along the coasts of the Gulf of Mexico and Florida (Fig. 5e). This indicates that the rate of change in near-surface humidity during the local onset of the low-pressure system is weaker than during the recovery phase. In other regions, cyclones display a more symmetrical behavior, although some grid points in the Tropical North Pacific show notable positive and negative values of $q_{asym}$. The model ensemble-mean’s bias of $q_{asym}$, denoted $\bar{q}_{asym}^{bias}$, are highest in regions where ERA5 exhibits the highest values of $q_{asym}$, except for the tropical North Atlantic eastward of Cuba (Fig. 5f). Except for the Gulf of Mexico, the biases are always opposite in sign to the reanalysis values, indicating that the models tend to underestimate the *asymmetry* of the humidity response to storms.

**Fig. 6:**
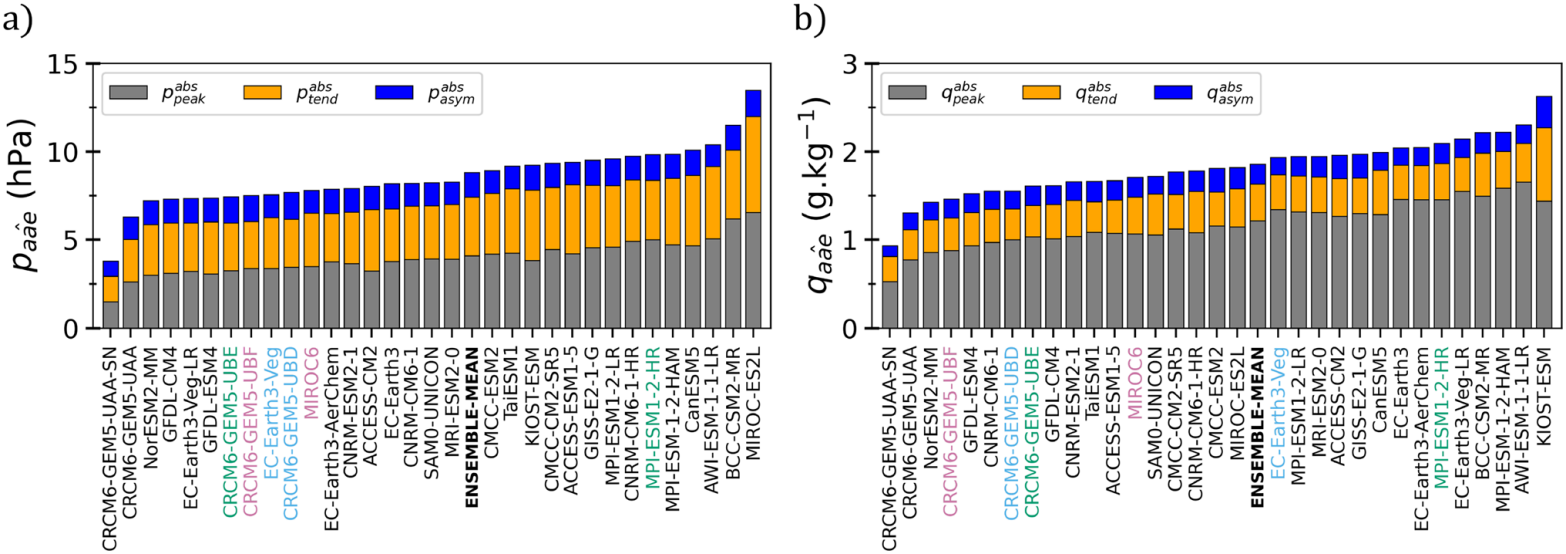
Additive absolute error ($\widehat{aae}$) of pressure anomalies (a) and near-surface humidity (b) for each individual model calculated using the linear approximation, and ranked from the smallest to the largest. The additive absolute error is the sum of the absolute error of the parameters *peak* (grey color), *tendency* (orange color), and *asymmetry* (blue color). Values are obtained by averaging over the entire North American domain. Regional simulations driven by a GCM and their corresponding driving GCM are labeled using the same color

Figure 6 displays the additive absolute error of the pressure anomaly ($p_{\widehat{aae}}$) and the near-surface humidity ($q_{\widehat{aae}}$) for each individual model over the entire North American domain, along with the decomposition of the error for each parameter. These errors were computed using Eq. 4 and averaged across the entire North American domain. The $\widehat{aae}$ metric does not evaluate the individual biases of each parameter in terms of overestimation or underestimation, but rather offers a measure of the overall error without allowing for potential compensatory effects between coefficients.

Figure 6a reveals that most models exhibit $p_{\widehat{aae}}$ values between $7\;\textrm{hPa}$ and $10\;\textrm{hPa}$. Notably, only the two regional simulations driven by ERA5 display $p_{\widehat{aae}}$ values below $7\;\textrm{hPa}$, with the simulation using spectral nudging achieving the lowest error, below $4\;\textrm{hPa}$. The model ensemble-mean shows a value of $p_{\widehat{aae}}$ close to $9\;\textrm{hPa}$, more than two times greater than the mean absolute error $p_{mae}$ ($3.9\;\textrm{hPa}$, recall Fig. 2a). This highlights the need to decompose the error introduced by the linear approximation in order to clarify the effects of compensation between parameters. Thirteen out of the 32 simulations show a pressure anomaly error below $8\;\textrm{hPa}$. These include CNRM-ESM2-1, all five simulations from the regional CRCM6-GEM6 model, three versions of EC-Earth3, both GFDL models, MIROC6, and NorESM2-MM. Interestingly, the three regional simulations driven by GCMs exhibit similar errors, despite the driving GCMs showing widely varying errors, with MPI-ESM1-2-HR reaching nearly $10\;\textrm{hPa}$ and ranking as the sixth worst model on this metric. The models with the highest errors, all exceeding $10\;\textrm{hPa}$, are AWI-ESM-1-1-LR, BCC-CSM2-MR, CanESM5, and MIROC-ES2L.

Figure 6a also shows the contribution of each error term ($p_{peak}^{abs}$, $p_{tend}^{abs}$, and $p_{asym}^{abs}$) to the absolute additive error metric. For most models, the highest contributions is observed from the $p_{peak}^{abs}$ term, with the exceptions being ACCESS-CM2 and KIOST-ESM. All simulations exhibit $p_{asym}^{abs}$ values ranging between $1\;\textrm{hPa}$ and $2\;\textrm{hPa}$, except for CRCM6-GEM5-UAA-SN with a value below $1\;\textrm{hPa}$. This parameter consistently has the smallest errors, as expected due to the low values of $p_{asym}$ (recall Fig. 4e). Overall, a consistent pattern is observed across all models and simulations: a relatively small error in the *asymmetry* parameter, a dominant error in the *peak* parameter, and a slightly lower error in the *tendency* parameter.

The CRCM6-GEM5-UAA-SN simulation also exhibits the lowest $q_{\widehat{aae}}$, with values below $1\,\mathrm {g\,kg^{-1}}$, whereas KIOST-ESM records the highest $q_{\widehat{aae}}$, with values $>2.5\,\mathrm {g\,kg^{-1}}$ (Fig. 6b). All other models and simulations show $q_{\widehat{aae}}$ errors between $1\;\text {and}\;2.5\,\mathrm {g\,kg^{-1}}$. The model ensemble-mean shows a value of $q_{\widehat{aae}}$ close to $1.9\,\mathrm {g\,kg^{-1}}$, which is substantially greater than the $1.1\,\mathrm {g\,kg^{-1}}$ of $q_{mae}$ (recall Fig. 2b). Furthermore, a similar pattern of additive error is observed with the magnitude of pressure anomaly errors. The three regional simulations driven by GCMs show similar performance and rank among the lowest $q_{\widehat{aae}}$ values, consistently outperforming their respective driving GCMs across all parameters. Other models such as NorESM2-MM, GFDL-ESM4, and CNRM-CM6-1 also demonstrate good performance on this metric. The error distribution for near-surface humidity differs from that of pressure anomalies, with $q_{peak}^{abs}$ contributing the largest portion of total error, while $q_{tend}^{abs}$ errors is two to three times lower, never exceeding $0.5\,\mathrm {g\,kg^{-1}}$ (except for KIOST-ESM). Absolute errors in the *asymmetry* parameter are also low for the near-surface humidity, typically below $0.3\,\mathrm {g\,kg^{-1}}$ in most models, again with KIOST-ESM as the outlier. Consequently, the absolute error of the *peak* parameter determines the overall performance of the $\widehat{aae}$ for near-surface humidity.

By combining the two variables, the ranking of all models based on the $\widehat{aae}$ highlights the same three simulations as the top performers for both pressure anomalies and near-surface humidity. These are the two regional simulations driven by ERA5 and NorESM2-MM. Models from GFDL also rank among the best, along with the regional simulations and, to a lesser extent, MIROC6 and CNRM-ESM2-1. Conversely, three models–BCC-CSM2-MR, AWI-ESM-1-1LR, and MPI-ESM-1-2-HAM–stand out for exhibiting high $\widehat{aae}$ for both variables. Similarly, albeit to a lesser extent, MIROC-ES2L, CanESM5, MPI-ESM1-2-HR and KIOST-ESM also demonstrate poor performance.

### Eulerian cyclones in specific regions

To assess the performance of CMIP6 and CRCM6 simulations, four regions with notable ensemble-mean biases were selected (Figure S1): the northern North Atlantic (NNA), western British Columbia (WBC), northern Quebec (NQC), and the Texas–Louisiana Gulf Coast (TLC). These regions were chosen for their distinct Eulerian cyclone characteristics relative to observations and to allow regional averaging while minimizing cancellation from opposing grid-point values. Each region covers 4 grid points zonally and 3 meridionally, roughly 600 km $\times$ 450 km.

#### Northern North Atlantic (NNA) region

**Fig. 7:**
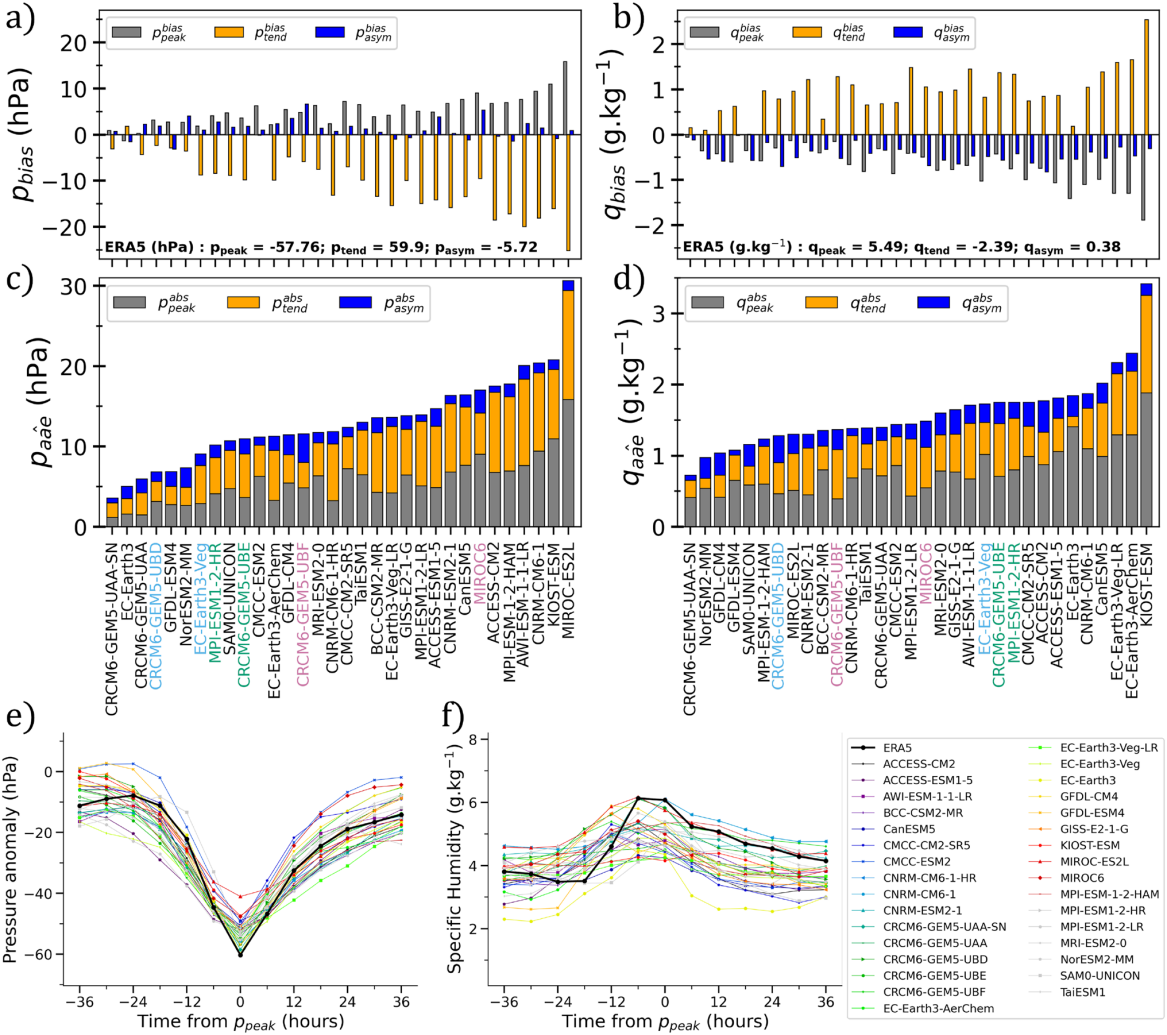
Bias (model - ERA5) of the parameters *peak* (grey color), *tendency* (orange color), and *asymmetry* (blue color) for the pressure anomaly (a) and the near-surface humidity (b) for each individual model calculated using the linear regression. (c) Additive absolute error ($\widehat{aae}$) of pressure anomalies and (d) near-surface humidity for each individual model calculated using the linear approximation, and ranked from the smallest to the largest. The additive absolute error is the sum of the absolute error of the three parameters. Regional simulations driven by a GCM and their corresponding driving GCM are labeled using the same color. Values are obtained by averaging over the region NNA shown in Figure S1. (e) Mean sea level pressure anomalies and (f) associated near-surface humidity, averaged across the strongest 7 storms between 1980 and 2014, 36 h before and after the passage of the storm at a random grid point within the region NNA of Figure S1

The NNA region experiences some of the most intense cyclonic systems and highest rates of pressure change along with a pronounced *asymmetry*, as reflected in Fig. 7a (see values in the panel) and Fig. 4. Figure 7a shows that all models, except EC-Earth3, display a positive bias for $p_{peak}$ and a negative bias for $p_{tend}$. Time series of pressure anomalies at a selected grid point within the region (Fig. 7d) reveal that most models fail to replicate the pronounced *peak* in pressure anomaly observed in ERA5. In particular, ERA5 data indicate a rapid decrease in *p* beginning approximately 18 h before the *peak* anomaly, a feature poorly captured by most models. ERA5 reaches a *peak* pressure anomaly of $-57.8\;\textrm{hPa}$ at $t_0$, whereas the models exhibit an average value that is $5.6\pm 3.2\;\textrm{hPa}$ higher. Consequently, $p_{tend}$ is underestimated in the models due to the physical linkage between the *peak* value and the rate of change. The relatively high $p_{asym}$ value from ERA5 in this region can be attributed to the steeper pressure decline preceding the *peak* compared to the rate of recovery after the *peak*.

Although the *peak* humidity observed during the most intense cyclones in this region is relatively low due to the cold SSTs, relatively high rates of near-surface humidity change are recorded, along with low values of *asymmetry* (see values in Figs. 7b and 5).

None of the models capture the magnitude of *peak* humidity observed in ERA5 during the passage of the strongest ETCs, resulting in a robust negative bias for $q_{peak}$ ($\bar{q}_{peak}^{bias}=-0.7\pm 0.4\,\mathrm {g\,kg^{-1}}$; Fig. 7b). Conversely, all models exhibit a compensating positive bias for $q_{tend}$. The intensification of pressure anomalies, which begins around $t-18$, coincides with a rapid increase in humidity time series. However, models fail to consistently replicate this behavior during the cyclone’s arrival phase, contributing to the observed bias in $q_{asym}$, despite lower apparent biases in the ridging phase (Fig. 7f).

Overall, regarding the absolute additive error for the three pressure parameters, EC-Earth3, GFDL-ESM4, NorESM2-MM, and the regional model CRCM6-GEM5 (driven by ERA5 and EC-Earth3-Veg) demonstrate the best performance in the NNA region (Fig. 7c). For near-surface humidity, the models GFDL-ESM4, GFDL-CM4, NorESM2-MM, and CRCM6-GEM-UAA-SN exhibit the lowest errors (Fig. 7d). NorESM2-MM emerges as the overall best-performing model when considering the absolute additive error across all six parameters, aside from the expected strong performance of CRCM6-GEM-UAA-SN. Notably, all regional simulations driven by GCMs outperform their respective driving GCM, except for CRCM6-GEM5-UBE, which shows slightly higher errors than MPI-ESM1-2-HR for pressure anomalies.

#### British Columbia (WBC) region

**Fig. 8:**
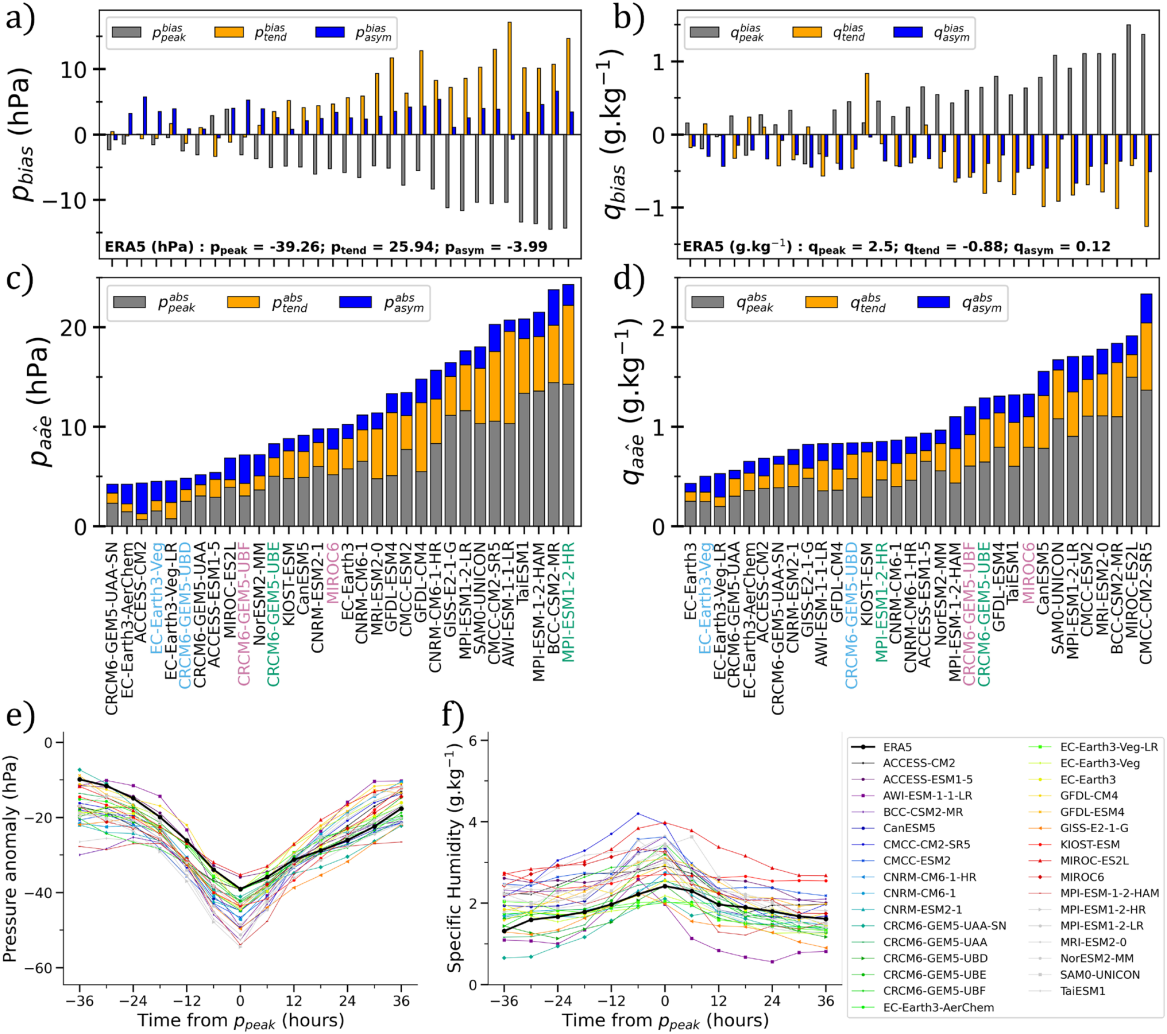
As in Fig. 7 but for the region WBC shown in Figure S1

In the WBC region, most models exhibit negative biases for the *peak* parameter and positive biases for the *tendency* parameter, which are opposite in sign to those found in the NNA region (Fig. 8a). The models that display the largest bias for $p_{peak}$ also tend to exhibit the largest bias for $p_{tend}$. Pressure anomalies for ERA5 in this region exhibit noticeable asymmetric intensification and recovery phases and the model ensemble-mean for $p_{asym}$ show values of similar magnitude to ERA5 but with an opposite sign, suggesting that the asymmetric signature of cyclones in this region is not accurately represented in the model simulations (Fig. 4e). Twenty-nine models out of the thirty-two have a positive bias for $p_{asym}$, with a model ensemble-mean bias of $3.2\pm 1.7\;\textrm{hPa}$ , compared to the ERA5 value of $-4.0\;\textrm{hPa}$. In a random grid point taken within the WBC region, most models show a symmetric pressure anomaly time series, while ERA5 displays a significantly asymmetric pattern (Fig. 8e). ERA5 pressure anomaly value at $t-36h$ is one of the highest compared to the models, but it averages out at $t+36$: the synoptic conditions three days prior to the *peak* anomaly reveal a background field characterized by strong early pressure anomaly values with models, which is less prominent with ERA5. This discrepancy underlies the observed *asymmetry* values and the models’ associated bias, which primarily arises from discrepancies during the recovery phase. Consequently, in this region, the bias in the *tendency* term remains small for the most accurate models (Fig. 8a).

All models except five exhibit a *peak* of humidity higher than that of ERA5 in the WBC region (Fig. 8b), along with excessive moisture content before $t_0$, as shown in Fig. 8e. Subsequently, the parameter $q_{tend}$ exhibits a notable negative bias across the majority of models. A similar interpretation to that of the pressure anomaly can be applied in this case: the higher background moisture levels observed during the onset of the storms tend to average out following their passage. Consequently, the *asymmetry* parameter is biased towards negative values for all models ($\bar{q}_{asym}^{bias}=-0.4\pm 0.1\,\mathrm {g\,kg^{-1}}$).

In the WBC region, most models simulate storms with excessive moisture and stronger pressure anomalies than ERA5, in contrast to the NNA region. A highly biased prestorm environment largely explains the systematic model biases across all parameters. Model spread is substantial: ACCESS-CM2 and EC-Earth3-family models perform best, with consistently low errors (Fig. 8c,d). Consequently, the regional simulation driven by EC-Earth3-Veg (CRCM6-GEM5-UBD) has the lowest error (after the ERA5-driven regional simulation), though slightly higher than its driving GCM. Conversely, MPI-ESM1-2-HR shows the highest pressure anomaly error, but its regional counterpart (CRCM6-GEM5-UBE) performs much better, with an error three times smaller. For near-surface humidity, only MIROC6 performs worse than its corresponding CRCM6 simulation. Overall, BCC-CSM2-MR, CMCC-CM2-SR5, and MPI-ESM1-2-LR are the weakest in this region.

#### Northern Quebec (NQC) region

The NQC region, located between Hudson Bay and Hudson Strait, is predominantly influenced by low-pressure systems that occur during both winter and fall (Fig. 3). This region exhibits one of the highest pressure anomalies and rate of pressure change on the North American continent, fairly well captured by models (Fig. 4). However, this region is particularly notable for exhibiting a positive *asymmetry* ($p_{asym}=2.4\;\textrm{hPa}$) that models fail to replicate, instead showing a strong negative bias.

**Fig. 9:**
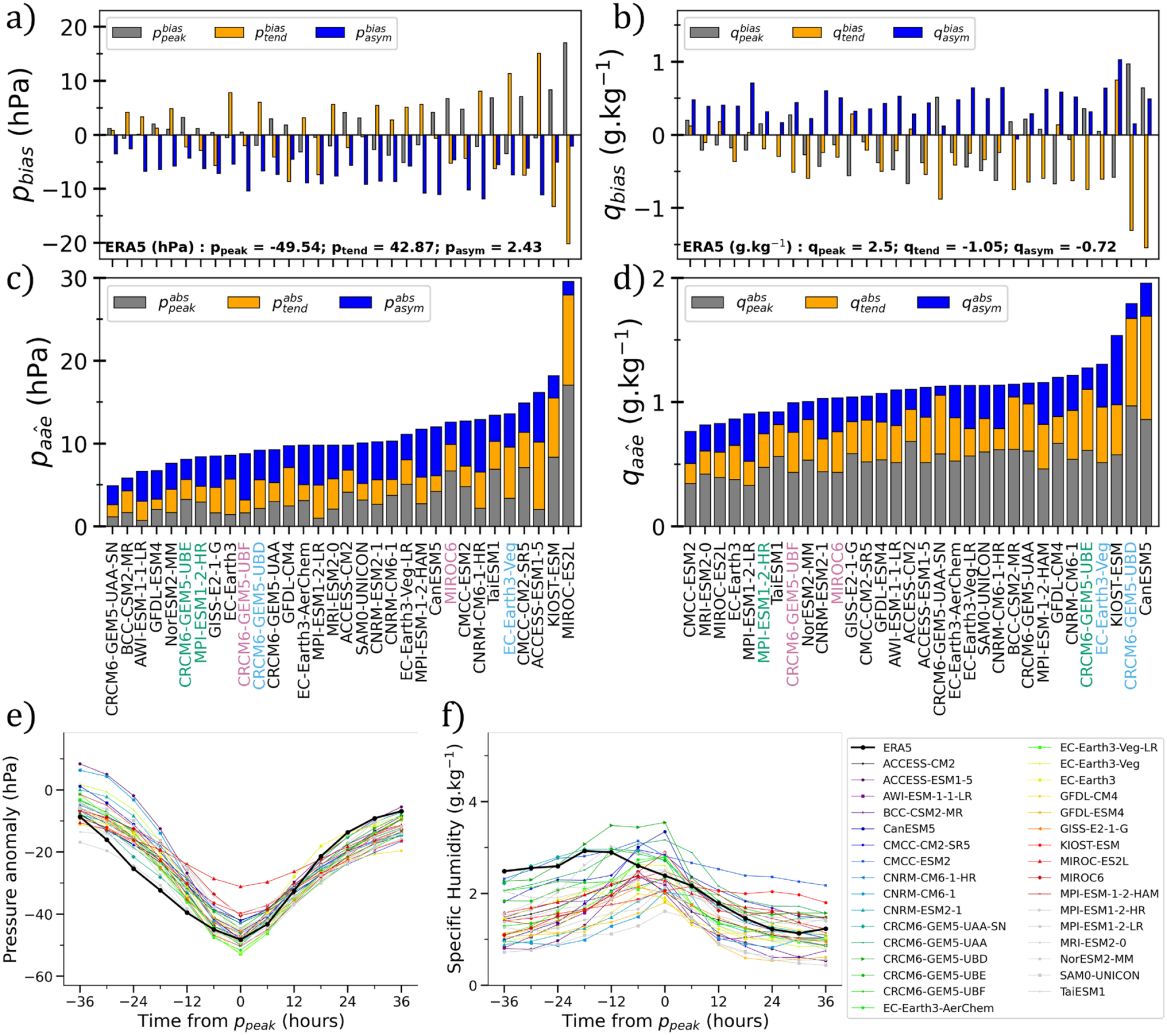
As in Fig. 7 but for the region NQC shown in Figure S1

As illustrated in Fig. 9a, biases in the *peak* and *tendency* parameters are relatively small in this region, with a few exceptions (e.g., MIROC-ES2L, KIOST-ESM, and ACCESS-ESM1-5). For the *asymmetry* parameter, all models exhibit a strong negative bias ($\bar{p}_{asym}^{bias}=-7.2 \pm 2.5\;\textrm{hPa}$), and the time-series plot in Fig. 9e reveals that models do not accurately capture both the deepening and recovery phases of the pressure field compared to observations. While ERA5 pressure anomalies show a gradual deepening followed by a rapid recovery after reaching the minimum, most models display the opposite behavior. Consequently, the bias for $p_{tend}$ remains low despite inaccuracies in capturing the two phases.

Similar to the pressure anomaly, models show a notable bias for the *asymmetry* parameter of near-surface humidity in the NQC region (Fig. 5f and Fig. 9b). This can be attributed to the relatively flat shape of the ERA5 humidity time series prior to $t_0$, showing no distinct peak at $t_0$ (Fig. 9f). In contrast, most models exhibit a more pronounced peak, leading to a relatively small bias in $q_{peak}$ despite their underestimation of moisture content before storm onset. Nevertheless, the recovery phase is generally well captured by the models.

The asymmetric signature in ERA5, which models fail to replicate for both variables, is of particular interest in this region. The prestorm environment likely contributes: in ERA5, near-surface humidity approaches saturation up to three days before the *peak* pressure anomaly, while models show drier conditions, leaving more room for increase. Additionally, local pressure evolves differently in ERA5 and the models: most models show a sharp drop before $t_0$ and a slower recovery, while ERA5 shows a more gradual drop but a sharper rise after $t_0$.

NorESM2-MM, MPI-ESM1-2-HR, and EC-Earth3 perform well in the region, in contrast to KIOST-ESM and EC-Earth3-Veg (Fig. 9c,d). Regional simulations show good skill for *p*, with the three GCM-driven simulations outperforming their respective driving GCMs. In contrast, regional simulations perform poorly for humidity, even when driven by ERA5. Notably, CRCM6-GEM5-UBE shows a marked degradation in humidity performance compared to its driving GCM, MPI-ESM1-2-HR.

#### Coastal Gulf of Mexico (TLC) region

The TLC region is situated close to the tropics and shows among the highest rate of near-surface humidity on land in North America. Some grid points in this region are significantly influenced by low-pressure systems occurring during the fall season, possibly due to the landfall of tropical cyclones (Fig. 3).

**Fig. 10:**
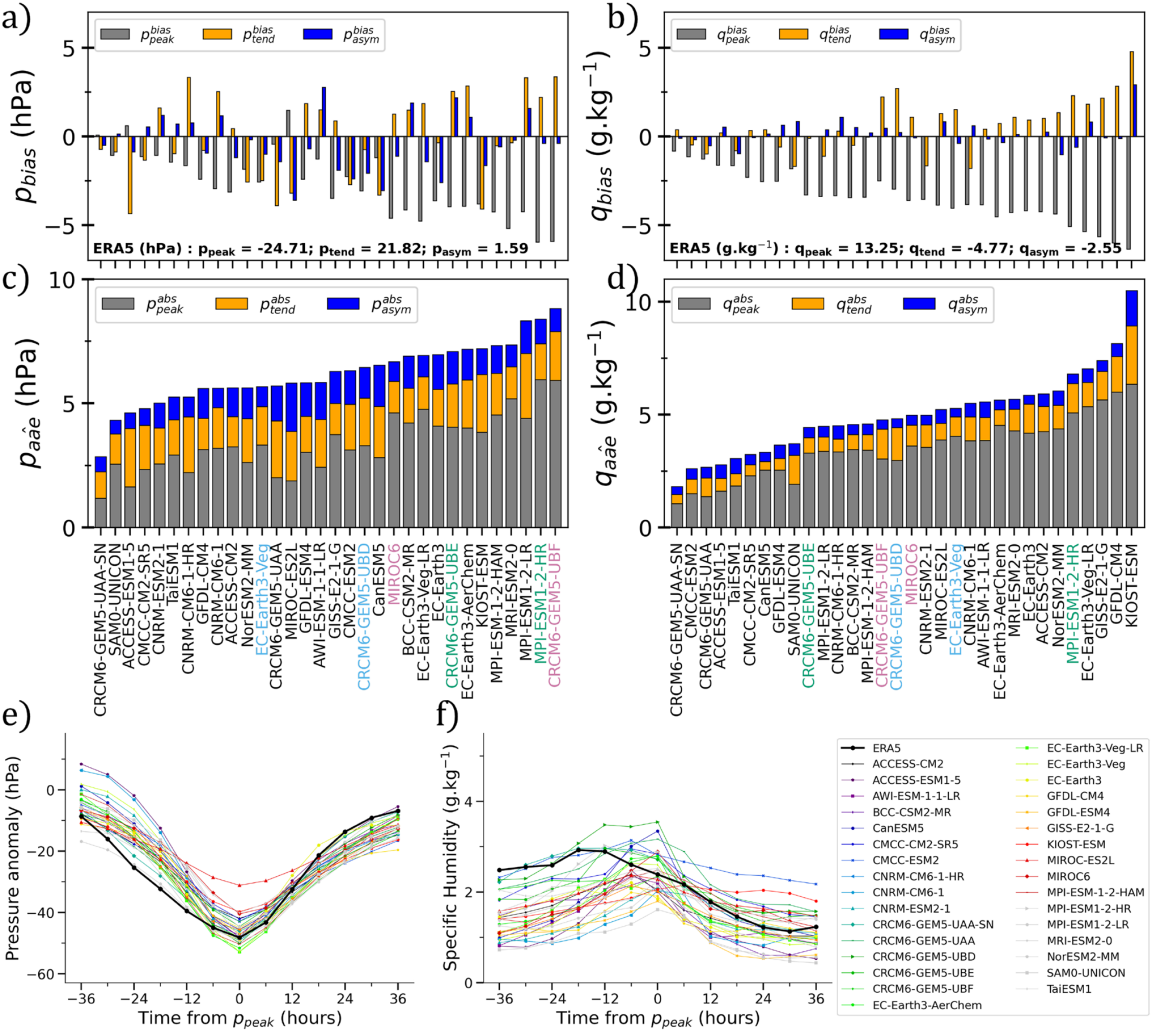
As in Fig. 7 but for the region TLC shown in Figure S1

Overall, with a few exception, models demonstrate reasonable performance in representing comparable pressure anomaly time-series with ERA5 (Fig. 10a). Only a slight negative bias for $p_{peak}$ can be noted.

The relatively high value of $q_{asym}$ for ERA5 in this region can be explained by examining the time series at a random point within the region (Fig. 10f): the high humidity in the grid box remains nearly constant during the early stage of storm development but drops rapidly after the storm peak. Models show only a small bias for $q_{tend}$ and $q_{asym}$, as most are able to replicate the 72-hour time series, although with a consistent negative offset in humidity content compared to ERA5. Indeed, none of the models reaches the humidity level observed in ERA5 at $t_0$ (Fig. 10b).

The primary challenge faced by models in this region is their inability to capture the high humidity levels observed during the passage of low-pressure systems, particularly prior to $t_0$, coupled with a systematic underestimation of the pressure anomaly. Similar to the behavior observed in the NQC region, the near-surface humidity time series from models exhibit a peak-shape that ERA5 does not replicate, likely because the specific humidity during the storm onset is already near saturation.

The regional simulation driven by ERA5 with spectral nudging (CRCM6-GEM5-UAA-SN) stands out as the best performer in terms of additive absolute error for both variables (Fig. 10c,d). All regional simulations reduce the near-surface humidity error compared to their driving GCMs and exhibit good skill in this regard. However, for the pressure anomaly error, two of them fail to show any noticeable improvement over their respective GCMs. Among the GCMs, SAM-UNICON, ACCESS-ESM1-5, CMCC-CM2-SR5, and TaiESM1 demonstrate consistently strong performance in this region, while KIOST-ESM, EC-Earth3-Veg-LR, and MPI-ESM1-2-HR rank among the weakest performers.

### Influence of the resolution

The performance of the models across the metrics used in this study may depend on various factors, with horizontal resolution likely to be a key determinant. Numerous studies have highlighted that increasing horizontal resolution can improve the overall performance of models in simulating ETCs (e.g., Andrade Cardoso et al. 2025; Jiaxiang et al. 2020). To further investigate this, models are grouped into four categories. The Regional Climate Model category (denoted with an "R") includes RCM simulations driven by GCM data (i.e., excluding both RCM simulations driven by ERA5 data). The High (H), Middle (M), and Low (L) horizontal resolution categories are defined using one third of the CMIP6 models, ranked according to their horizontal resolution. Table 1 specifies which models belong to each category. In addition, to better assess the added value of the regional simulations relative to their driving GCMs, a "D" category grouping the three driving GCMs is introduced.

**Fig. 11:**
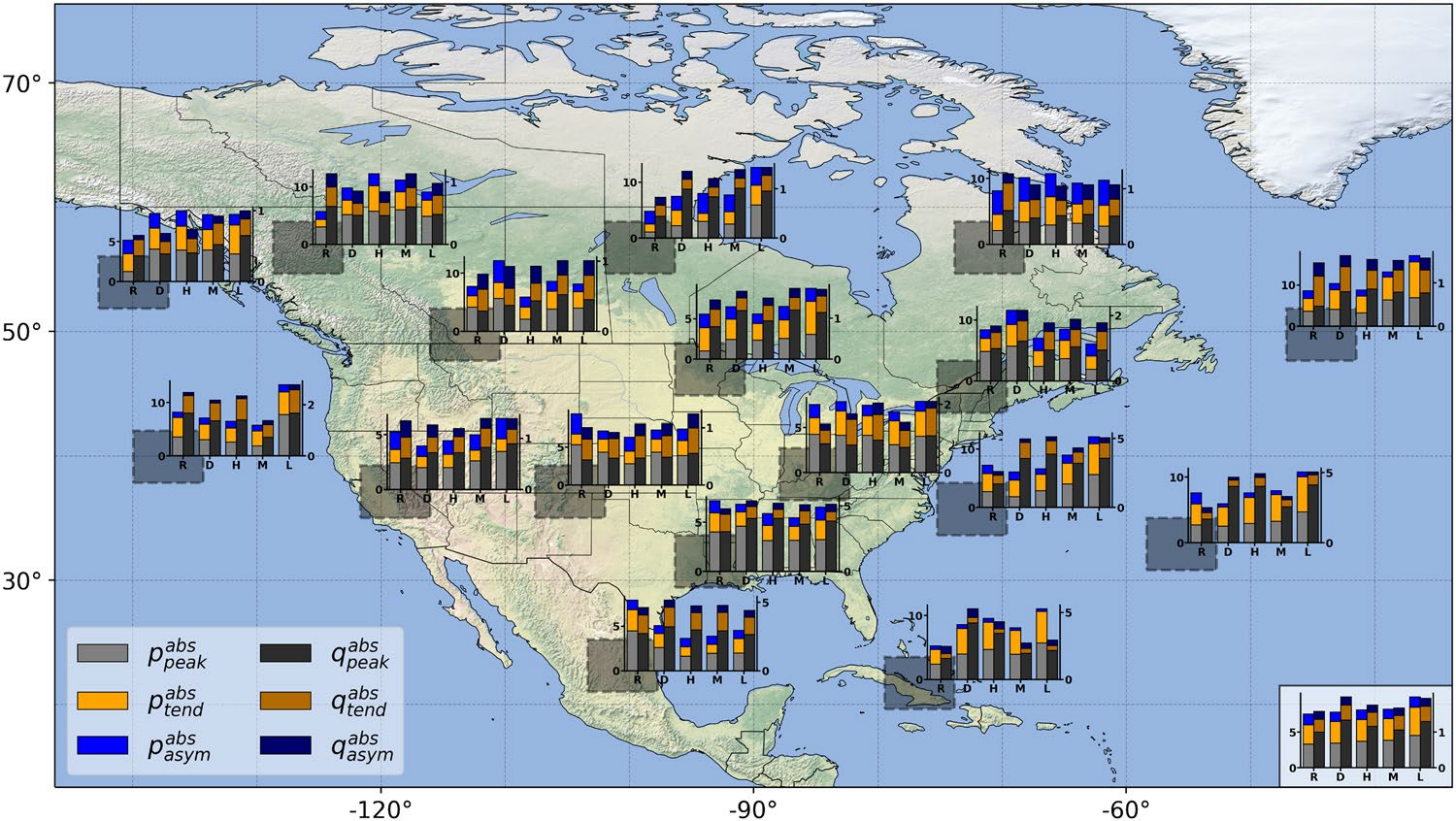
The median of the additive absolute error ($\widehat{aae}$) for each group of models categorized by their horizontal resolution across different regions: R denotes the three GCM-driven regional simulations, D the corresponding three driving GCMs, and H, M, and L the high-, middle-, and low-resolution model groups, respectively. The regions are delineated by the grey squares in the lower left of each bar plot and are of equal size (4$\times$3 grid cells). The inset in the lower right corner displays the $\widehat{aae}$ for the entire North American domain

Figure 11 presents the median of the additive absolute error ($\widehat{aae}$) for the three parameters (*peak*, *tendency*, and *asymmetry*), computed across models within each of the five categories and across various regions in North America. All regions have equal size (4$\times$3 grid cells), and those discussed in Sect. 4.3 are also included. When averaged over the entire domain (bottom-right panel of Fig. 11), regional simulations outperform GCMs for both variables. However, this improvement does not hold consistently at the regional scale. No clear or uniform pattern emerges: models from a given resolution category that perform best for one variable in a region often do not for the other. Likewise, a resolution category that ranks well in one region may perform poorly in a nearby region. Among GCM categories, the Low-resolution models exhibit the highest errors, while the High- and Middle-resolution categories perform comparably, with Middle-resolution models showing slightly better accuracy for near-surface humidity. The inferior performance of Low-resolution models is evident region by region: although they do not always have the highest errors, they generally give the worst results by a small margin. In general, the *peak* parameter appears to be the primary driver of overall performance differences between categories, with the individual error in the other two parameters playing a secondary role. Nevertheless, regions above $45^{\circ }$N more frequently display large errors in the *asymmetry* parameter. At these latitudes, low-pressure systems show opposite-signed *asymmetry* depending on longitudes (Fig. 4e), a pattern that models struggle to replicate (Fig. 4f).

In all regions north of $45^{\circ }$N, except for one, the regional simulations exhibit the best performance for the additive error associated with pressure anomaly parameters. While regional simulations often inherit *asymmetry* errors from their driving GCMs within this part of the domain, they consistently reduce the *peak* error, which underlies their superior overall performance. In contrast, only a single region south of $45^{\circ }$N identifies the regional climate model category as the most accurate for pressure anomalies; at these latitudes, the asymmetry error is consistently higher in the regional simulations compared to their driving GCMs. For near-surface humidity, regional simulations show modest improvements relative to their driving GCMs across most regions of continental North America and the North Pacific. However, the added value is particularly pronounced in the humid North Atlantic region, where the model ensemble-mean exhibits a substantial negative bias in peak surface humidity (Fig. 5b). In this area, the regional simulations better represent moisture contributions in the lower atmosphere associated with low-pressure systems along the East Coast and do not inherit the biases in *peak* of their driving GCMs. Consistent with the regional-scale analysis (Sect. 4.3), errors in the regional simulations are not necessarily inherited from their driving GCMs.

Overall, when the additive absolute error for at least one of the two variables is large (e.g., $>10\;\textrm{hPa}$ for *p* or $>4\,\mathrm {g\,kg^{-1}}$ for *q* in any resolution category), regional simulations consistently provide a significant improvement over GCMs. This suggests that the CRCM6 simulations provide added value relative to their boundary conditions by accurately capturing both the cyclogenesis and cyclolysis phases along ETC track, as demonstrated by Poan et al. (2018) in the context of winter North American storm events.

## Discussion

In this paper, we evaluate how well climate model simulations represent the pressure anomaly and near-surface humidity around the most intense low-pressure systems over North America using an Eulerian perspective. To do so, we analyze three parameters that characterize the time series of pressure anomaly and humidity: the *peak* value, the mean *tendency* and the *asymmetry*. This framework enables a detailed assessment of the sources of error in climate models, relative to the ERA5 reanalysis, in capturing the temporal evolution of dynamical and thermodynamical fields associated with intense storms in North America.

The three-parameter decomposition is shown to be a great fit in most of the domain with the exception of certain areas of the tropical Atlantic, potentially along tropical cyclone tracks. Across the entire domain, the ensemble-mean of the model exhibits an additive absolute error for the three parameters of approximately $9\;\textrm{hPa}$, which is more than twice the mean absolute error of $3.9\;\textrm{hPa}$. This discrepancy underscores the presence of error compensation among the three parameters, which acts to reduce the overall mean error.

We found that the strongest *peak* and *tendency* pressure anomalies are generally larger in ERA5 compared to the model ensemble-mean over oceanic grid points, while they are smaller over continental areas (Fig. 4b,d). Several studies have shown that CMIP-type models simulate an overly zonal North Atlantic storm track, with extratropical cyclones not extending sufficiently poleward (Priestley et al. 2020; Zappa et al. 2013b), especially when considering coarse-resolution models. Priestley et al. (2023) highlighted that the insufficient poleward displacement of cyclones during winter in CMIP6 models is linked to a persistent cold anomaly south of Greenland in the North Atlantic. This underrepresentation of cyclones on the northern flank of the North Atlantic storm track, north of $40^{\circ }$N, likely explains the biases observed in this region. In addition, some of these biases, particularly regarding the *asymmetry*, may also originate from a misrepresentation of SSTs and their impact on storm intensification (Booth et al. 2012). Furthermore, Baker et al. (2022) showed that higher-resolution ($\sim 25~\textrm{km}$) simulations improve the representation of tropical cyclones undergoing extratropical transition compared to lower-resolution ( 100 km) models in the western North Atlantic. Our results further confirm that higher-resolution GCMs and the RCM perform better in both the western North Atlantic and northern North Pacific (Fig. 11). Moreover, the rapid deepening of cyclones observed in the northern North Atlantic with ERA5 (Fig. 7c) provides clear evidence of the frequent occurrence of explosive extratropical cyclones in this region. The models fail to capture this abrupt intensification, which is consistent with the findings by Seiler et al. (2018), who showed that CanESM2 (even when downscaled with CanRCM4) struggles to reproduce a sufficient number of explosive extratropical cyclones along the Atlantic coast of North America.

As in previous studies (Colle et al. 2013; Jiaxiang et al. 2020; Priestley et al. 2020; Seiler et al. 2018; Zappa et al. 2013b), we found that higher horizontal resolution for GCMs generally leads to better performance in simulating ETC characteristics (Fig. 11). Yet, this improvement appears modest when comparing high- and medium-resolution models, becoming substantial only when coarse-resolution models are considered. Furthermore, the benefits of higher resolution vary by region and variable. For instance, most continental areas show little or no improvement in pressure anomaly errors with higher resolution, and near-surface humidity errors exhibit minimal sensitivity to resolution across much of North America.

The two RCM simulations driven by ERA5 show the best overall performance across the domain and consistently rank among the best-performing simulations out of the 32 analyzed in each of the four regions, which is expected since errors are computed relative to ERA5. Moreover, spectral nudging consistently reduces errors; the ERA5-driven simulation incorporating it frequently stands out as an outlier with substantially lower errors, particularly for pressure anomaly. Overall, the three GCM-driven regional simulations also perform well for both variables and generally improve upon their driving data. However, regional differences remain: over much of continental North America south of $45^{\circ }$N, where GCMs exhibit relatively low errors, the GCM-driven simulations show little to no improvement, and in some cases even deterioration. In contrast, they provide substantial added value in regions frequently affected by intense low-pressure systems, such as the western and northern North Atlantic and over land north of $45^{\circ }$N. This is consistent with the findings of Poan et al. (2018) using an earlier CRCM version. The CRCM6-GEM5 typically adds value in regions where GCMs display substantial errors. Interestingly, the performance of the driving model does not appear to strongly influence the performance of the RCM. For example, CRCM6-GEM5 simulations often show similar performance despite being driven by GCMs with varying levels of skill.

The biases and errors calculated in this paper may result from several factors that are difficult to decipher within the Eulerian framework used (Bernardino et al. 2009): (i) discrepancies in the position and intensity of storm tracks between the models and ERA5 (Priestley et al. 2020; ii) differences in propagation speed of the systems, as shown by Crawford et al. (2023; iii) variations in the maturity of low-pressure systems affecting each region (Dacre et al. 2019); and (iv) disparities in diabatic processes (Ma et al. 2017; Willison et al. 2013), among others. However, our methodology does not allow us to isolate the specific origins of these errors. Nevertheless, local pre-storm conditions appear to play a crucial role in the analyzed regions, contributing significantly to model errors by influencing all three parameters. Regional bias patterns may also reflect a seasonal modulation by surface conditions poorly represented in GCMs. A notable illustration is found in the Great Lakes, where the lakes exert a strong, season-dependent influence on passing low-pressure systems (Notaro et al. 2013; Xiao et al. 2018). As shown in Fig. 3, this results in a complex spatial mosaic of dominant seasons across the region. This region also coincides with localized bias patterns in various parameters (Figs. 4 and 5), suggesting that the crude representation of the Great Lakes in CMIP-class GCMs (Briley et al. 2021) may contribute to the regional bias signatures observed in our results, as well as limitations in representing ETC characteristics over regions with strong air–lake contrasts (Poan et al. 2018).

Some limitations of the study should be acknowledged. A key one lies in the use of ERA5 as a reference, as discussed in Sect. 3.3. Another limitation relates to the choice of variables and the way the most intense cyclones are selected. The 35-event time series of near-surface humidity per grid point, selected based on the strongest pressure anomalies for each year between 1980 and 2014, exhibit substantial variability (e.g., Fig. 1b,d). Even when considering the composite of the seven most intense low-pressure systems, the resulting average remains subject to uncertainty driven by internal variability. This limitation warrants particular caution when interpreting direct model-to-model comparisons, while such variability is partially mitigated when using model ensembles. Moreover, the 6-hourly temporal resolution may be insufficient to accurately capture the deepening and recovery phases of both *p* and *q*, as well as their respective *peak* values. A finer temporal resolution would enable a more detailed analysis of the local evolution of the most intense systems. Furthermore, the three parameters used in this study, along with their associated biases and the definition of the time window (3 days), are to some extent interdependent. For example, a higher *peak* value is typically associated with a higher *tendency* (see Fig. 2a vs. c), as they are physically linked. This interdependence implies that a bias in one parameter can disproportionately affect the composite error metric $\widehat{aae}$, potentially overstating the total error.

## Conclusion

In this paper, we evaluated the ability of 32 simulations, performed using 27 CMIP6 global climate models and one regional climate model, at representing the evolution of surface pressure anomalies and the associated near-surface humidity during the passage of strong cyclonic events in North America. The evaluation was performed using an innovative two-part methodology: first, we apply a novel Eulerian-based approach to identify the most intense low-pressure systems at each grid point across North America; second, we characterize the temporal evolution of each event using a three-parameter decomposition of its cyclonic signature. The three parameters include the *peak* value, the mean *tendency*, and the *asymmetry* of the signature. This evaluation was performed using the ERA5 reanalysis as the reference.

Our method offers a novel framework for characterizing model biases in simulating local dynamical and thermodynamical conditions associated with the passage of intense low-pressure systems over North America. The three-parameter decomposition effectively captures key regional differences in intense cyclonic systems, including cyclone depth, intensification rates, and asymmetry in their temporal structure.

Our findings suggest that GCMs often suffer from similar biases in characterizing local environmental changes associated with these systems; however, the origins of these biases vary by region. While model resolution does affect error magnitude, its impact is less prominent over continental areas. In regions with high track density or frequently affected by explosive extratropical cyclones (e.g., the western and northern North Pacific), higher resolution reduces pressure anomaly errors, whereas near-surface humidity shows little sensitivity with no consistent resolution-related pattern.

Simulations using the CRCM6-GEM5 model at 12-km resolution generally exhibit the smallest errors across the entire domain. Notably, their performance remains consistent regardless of the driving boundary conditions, whether from ERA5 or three different CMIP6 models, suggesting that the choice of driving data has limited influence on the errors. Regional simulations show particular strength north of $45^{\circ }$N and over oceanic regions, where they offer clear added value relative to GCMs, especially where GCM errors are large. Nonetheless, substantial errors persist in some regions.

Future work could integrate our Eulerian detection method with an object-based tracking approach to further elucidate the reasons behind these biases in each region through Lagrangian statistics, as well as extend the framework to a seasonal decomposition of the analysis to better account for the seasonal variability in the characteristics of the strongest low-pressure systems.

## Supplementary Information

Below is the link to the electronic supplementary material.

Supplementary file 1 (pdf 1650 KB)

## Data Availability

All global climate model output data are available from the Earth System Grid Federation https://esgf-node.ipsl.upmc.fr/search/cmip6-ipsl/. ERA5 data are available from https://cds.climate.copernicus.eu/datasets. CRCM6-GEM5 data are available from the authors upon reasonable request.

## Code availability

The Python scripts used to conduct the analysis are available at https://github.com/viroki7/De_Meyer_etal_2025_Eulerian_Storms.git
