# Supplementary material for: An Eulerian evaluation of intense low-pressure systems over North America in CMIP6 and a regional climate model
Source: Clim Dyn. 2026 Jun 26;64(7):306. doi: 10.1007/s00382-026-08260-7 (PMC13309517; doi:10.1007/s00382-026-08260-7)
Supplement: Supplementary file 1 — (pdf 1650 KB) [file 382_2026_8260_MOESM1_ESM.pdf]

Simulation Name	Model	Dynamics Core	Driving Data	Spectral Nudging	Lowest Hybdrid Level for Nudging	Microphysics Scheme	Deep Convective Scheme	Lake model	Land Surface Scheme	Geophysical Fields	Domain (CORDEX)	Time Steps	Resolution	Vertical Levels	Use of TOFD scheme
**CRCM6-GEM5-UAA-SN**	CRCM6	GEM5.1.1	ERA5	U,V,T	0.85	P3	Kain–Fritsch	FLake	CLASS3.6	CCI	NAM-11	5min	0.11°	71	Yes
**CRCM6-GEM5-UAA**	CRCM6	GEM5.1.1	ERA5	None	None	P3	Kain–Fritsch	FLake	CLASS3.6	CCI	NAM-11	5min	0.11°	71	No
**CRCM6-GEM5-UBD**	CRCM6	GEM5.1.1	Ec-Earth3-Veg	U,V	0.7	P3	Kain–Fritsch	FLake	CLASS3.6	LANDCOVER V2	NAM-11	5min	0.11°	71	Yes
**CRCM6-GEM5-UBE**	CRCM6	GEM5.1.1	MPI-ESM1-2-HR	U,V	0.7	P3	Kain–Fritsch	FLake	CLASS3.6	LANDCOVER V2	NAM-11	5min	0.11°	71	Yes
**CRCM6-GEM5-UBF**	CRCM6	GEM5.1.1	MIROC6	U,V	0.7	P3	Kain–Fritsch	FLake	CLASS3.6	LANDCOVER V2	NAM-11	5min	0.11°	71	Yes

Table A1: Regional model configuration for all simulations. TOFD stands for Turbulent Orographic Form Drag.

Appendix A Supplementary Material

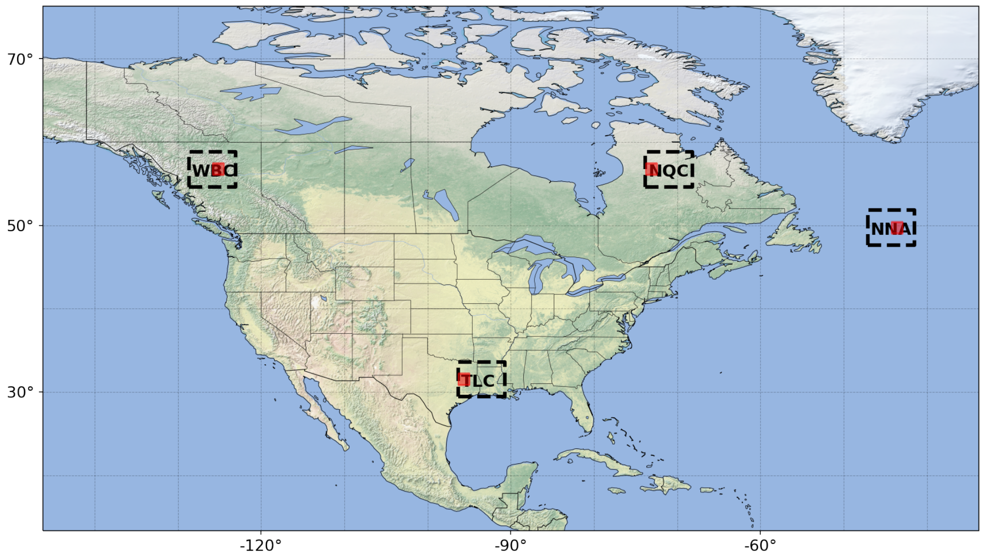

Fig. S1: Geographical boundaries of the domains selected for the regional analysis. The red squares correspond to the randomly chosen grid points chosen to display the time series of the different models.
